# Studies on anti-rabphilin-3A antibodies in 15 consecutive patients presenting with central diabetes insipidus at a single referral center

**DOI:** 10.1038/s41598-022-08552-y

**Published:** 2022-03-15

**Authors:** Zenei Arihara, Kanako Sakurai, Satsuki Niitsuma, Ryota Sato, Shozo Yamada, Naoko Inoshita, Naoko Iwata, Haruki Fujisawa, Takashi Watanabe, Atsushi Suzuki, Kazuhiro Takahashi, Yoshihisa Sugimura

**Affiliations:** 1 Department of Endocrinology and Metabolism, National Hospital Organization, Sendai Medical Center, Sendai, Miyagi 983-8520 Japan; 2 Hypothalamic and Pituitary Center, Moriyama Neurological Center Hospital, Edogawa, Tokyo, 134-0081 Japan; 3 Department of Pathology, Tokyo Metropolitan Geriatric Hospital, Itabashi, Tokyo, 173-0015 Japan; 4 Department of Endocrinology and Diabetes, Daido Hospital, Nagoya, Aichi 457-8511 Japan; 5 Department of Endocrinology, Diabetes and Metabolism, Fujita Health University, Toyoake, Aichi 470-1192 Japan; 6 Department of Biochemistry, Fujita Health University, Toyoake, Aichi 470-1192 Japan; 7 Department of Endocrinology and Applied Medical Science, Tohoku University Graduate School of Medicine, Sendai, Miyagi 980-8585 Japan

**Keywords:** Neuroendocrine diseases, Pituitary diseases

## Abstract

Central diabetes insipidus (CDI) is a rare condition caused by various underlying diseases including inflammatory and autoimmune diseases, and neoplasms. Obtaining an accurate definitive diagnosis of the underlying cause of CDI is difficult. Recently, anti-rabphilin-3A antibodies were demonstrated to be a highly sensitive and specific marker of lymphocytic infundibuloneurohypophysitis (LINH). Here, we report a detailed case series, and evaluated the significance of anti-rabphilin-3A antibodies in differentiating the etiologies of CDI. A prospective analysis was conducted in 15 consecutive patients with CDI from 2013 to 2020 at a single referral center. Anti-rabphilin-3A antibodies were measured and the relationship between antibody positivity and the clinical/histopathological diagnoses was evaluated. Among 15 CDI patients, the positive anti-rabphilin-3A antibodies were found in 4 of 5 LINH cases, 3 of 4 lymphocytic panhypophysitis (LPH) cases, one of 2 sarcoidosis cases, and one intracranial germinoma case, respectively. Two Rathke cleft cyst cases and one craniopharyngioma case were negative. This is the first report of anti-rabphilin-3A antibodies positivity in CDI patients with biopsy-proven LPH. Measurement of anti-rabphilin-3A antibodies may be valuable for differentiating CDI etiologies.

## Introduction

Central diabetes insipidus (CDI) is a rare condition, with a reported prevalence of approximately 7–10 per 100,000 inhabitants^1^. CDI is caused by the destruction or degeneration of neurons originating in the supraoptic and paraventricular nuclei of the hypothalamus. The causes of CDI are tumors (such as germinomas and craniopharyngiomas), infiltrative diseases (such as Langerhans cell histiocytosis), neurosurgery, trauma, and, in rare cases, genetic defects in vasopressin synthesis^2–4^. However, up to 15% of CDI causes remain idiopathic^5–7^, although, Di Iorgi et al.^8^ showed idiopathic CDI is a very uncommon condition. An autoimmune process involving destruction of the neurohypophysis may be involved in many patients with idiopathic CDI^9,10^. Biopsy samples and postmortem examination of patients demonstrate lymphocytic infiltration of the pituitary stalk. Lymphocytic infundibuloneurohypophysitis (LINH) accounts for a substantial subset of autoimmune CDI cases and is characterized by lymphocytic inflammation of the posterior pituitary and infundibular stalk^2,4,11–17^. In addition, IgG4-related hypophysitis is a subtype of autoimmune hypophysitis associated with multiorgan IgG4-related systemic disease^18–23^. Pathological examination is required for a definitive diagnosis. However, surgery or biopsy of the pituitary is seldom performed because of invasiveness; therefore, most patients are diagnosed according to their clinical manifestations. Cranial magnetic resonance imaging (MRI) to identify hyperintensities in the posterior pituitary or thickening of the pituitary stalk can help determine the cause of CDI. Thickening of the pituitary stalk is a nonspecific finding, so some patients with pituitary stalk thickening later develop germinomas or histiocytosis^24,25^. Therefore, patients should undergo regular endocrine follow-up. Anti-vasopressin-cell antibodies have been detected in patients with idiopathic CDI; however, these antibodies have also been detected in DI of other etiologies, including Langerhans cell histiocytosis and germinomas, and thus cannot be considered a reliable marker of autoimmune-mediated CDI^26,27^.

Recently, anti-rabphilin-3A antibodies were shown to be a highly sensitive and specific diagnostic marker for LINH. In cases with a biopsy-proven diagnosis, the presence of anti-rabphilin-3A antibodies showed a sensitivity of 100% in diagnosing LINH in 4 of 4 patients with LINH, and a specificity of 100% in distinguishing sellar/suprasellar masses (34 patients including 18 CDI patients) that were difficult to differentiate from LINH in clinical practice^28^. In that study, samples from patients with various pituitary disorders were collected from several institutes in Japan and from Johns Hopkins University.

The aim of the present study is to clarify the significance of anti-rabphilin-3A antibodies in differentiating the etiologies of CDI. All patients presenting with polyuria and polydipsia underwent endocrinological tests, including the hypertonic saline infusion test, and MRI from 2013 to 2020. We evaluated anti-rabphlin-3A antibodies in consecutive CDI patients from a single referral center, in which the staff skillful in diagnosis and treatment of CDI was enrolled. This is the first case series to evaluate the presence of anti-rabphilin-3A antibodies in consecutive patients with CDI.

## Materials and methods

### Patients

The consecutive patients who were diagnosed with CDI at Sendai Medical Center (Sendai, Japan) from April 2013 to March 2020 were recruited. All of the patients with CDI were included, but the patients with CDI that developed as a complication of surgery were excluded from this study. They were admitted to our hospital because of polyuria and polydipsia. CDI was diagnosed according to the arginine vasopressin (AVP) responses on the hypertonic saline infusion test. Anterior pituitary function was also evaluated by the basal levels and/or responses of adrenocorticotropic hormone (ACTH), thyroid stimulating hormone (TSH)/ prolactin (PRL), growth hormone (GH) and luteinizing hormone (LH)/ follicle stimulating hormone (FSH) to corticotropin-releasing hormone (CRH), thyrotropin-releasing hormone (TRH), growth hormone-releasing hormone (GRH)/growth hormone-releasing peptide-2 (GHRP-2) and gonadotropin-releasing hormone (GnRH), respectively. In addition, the serum levels of IgG4 and various autoantibodies were measured.

All patients underwent imaging examinations, such as MRI of the brain and X-ray computed tomography of the whole body. MRI is useful for evaluation and follow-up of lesions^29^. Diffuse enlargement of the anterior pituitary with strong homogeneous contrast enhancement in the lesion is characteristic of lymphocytic adenohypophysitis^30–32^. Thickening of the pituitary stalk and enlargement of the neurohypophysis with gadolinium enhancement are observed in LINH^12^. The findings of both lymphocytic adenohypophysitis and LINH, such as whole pituitary gland swelling and pituitary stalk thickening, are observed in LPH^14,33^. In addition, ^67^Ga scintigraphy is performed in patients with suspected sarcoidosis.

All research was performed in accordance with guidelines of Japan Endocrine Society. The following criteria were used to diagnose LINH according to the clinical guidelines of Japan Endocrine Society^29^: (I) main symptoms: thirst, polydipsia and polyuria; (II) laboratory data and pathological findings: (1) laboratory findings that match the criteria of CDI, (2) enlargement of the pituitary gland or stalk on imaging, (3) strong and diffuse enhancement in the neurohypophysis and/or stalk lesion on MRI with gadolinium enhancement, and (4) cell infiltration consisting mainly of lymphocytes in the lesion in biopsy specimen; and (III) additional information: preserved anterior pituitary function in most cases and disappearance of the pituitary gland or stalk enlargement during the natural course in most cases. A definitive diagnosis of LINH is made when all items in I and II are fulfilled. A probable diagnosis of LINH is established when all items in I and items 1, 2, and 3 in II are fulfilled.

The following criteria were used to diagnose lymphocytic panhypophysitis (LPH): (I) main symptoms: symptoms due to a mass lesion in the pituitary gland or hypopituitarism and symptoms due to CDI; (II) laboratory and pathology findings: (1) decreased levels of one or more anterior pituitary hormones as well as those from the targeted organs, (2) decreased responses of anterior pituitary hormones in stimulation tests, (3) laboratory findings that match those of CDI, (4) diffuse enlargement of the pituitary gland and/or stalk on imaging, (5) homogenous and strong enhancement of the pituitary gland or stalk lesion on MRI with gadolinium enhancement, and (6) cell infiltration consisting mainly of lymphocytes in the pituitary gland or stalk in biopsy specimens. A definitive diagnosis of LPH is made when the all items in I and II are fulfilled. A probable diagnosis of LPH is made when all items in I and items 1–5 in II are fulfilled.

In addition, according to the clinical guidelines of the Japan Endocrine Society^29^, the differential diagnosis of primary hypophysitis is defined by the following criteria: (I) the systemic diseases sarcoidosis, granulomatosis with polyangiitis, Langerhans cell histiocytosis, syphilis, tuberculosis, mycoses and IgG4-related disease and (II) the sellar and parasellar diseases germinoma, Rathke cleft cyst, craniopharyngioma, pituitary adenoma, and chronic inflammation in parasellar lesions such as the paranasal or cavernous sinus.

Differential diagnosis among LINH, lymphocytic adenohypophysitis and LPH was conducted based on clinical manifestations, MRI findings and histopathological examination. The patients whose pituitary specimens were not available for histopathological examination were diagnosed according to the criteria of the Japan Endocrine Society^29^ or the Japan Society of Sarcoidosis and Other Granulomatous Disease 2015 diagnostic criteria and guidelines for sarcoidosis^34^.

All patients were examined for anti-rabphilin-3A antibodies. The patients provided informed consent for all the studies. This study was approved by the ethics committees of Sendai Medical Center(RIN31-32), Fujita Health University (HM17-91) and Nagoya University (1355).

### Hypertonic saline infusion test

Hypertonic saline infusion tests were performed after an overnight fast, but free water drinking. An indwelling catheter was inserted in the forearm vein at 8:00 am, followed by intravenous drip infusion of 5% saline at 0.05 mL/kg/min for 120 min. Blood samples were collected before and after the infusion. AVP assays were performed using the AVP-RIA kit Neo Mitsubishi from Mitsubishi Petrochemical Co. Ltd. (Tokyo, Japan) between April 2013 and August 2015 or the AVP kit Yamasa from Yamasa Corporation (Choshi, Japan) after September 2015.

### Measurement of anti-rabphilin-3A antibodies by Western blotting

The samples for measuring anti-rabphilin-3A antibodies were collected at the beginning of hypertonic saline infusion tests. Measurement of anti-rabphilin-3A antibodies in serum was performed as follows, at the Graduate School of Medicine, Nagoya University from April 2013 to March 2017 and at Fujita Health University after April 2017. A vector containing the full-length human rabphilin-3A gene was transfected into HEK293FT cells to produce a recombinant human rabphilin-3A protein. The vector was designed to incorporate a V5 tag onto the encoded rabphilin-3A protein. The rabphilin-3A gene was inserted into the N-terminus of the V5 tag (pcDNA3.1-rabphilin-3A-V5-His). The V5 expression depends on the start codon of rabphilin-3A. The expression of the recombinant rabphilin-3A protein was confirmed using an anti-V5 antibody. As a control, the same vector but without the rabphilin-3A gene was transfected into HEK293FT cells. Anti-rabphilin-3A antibodies in the serum were detected by Western blotting using the recombinant human rabphilin-3A protein lysate as the antigen and the serum as the primary antibody. A protein band presenting a size of 76 kDa appeared in the lysate of cells transfected with rabphilin-3A protein but not in that of control cells, which was considered to be positive for anti-rabphilin-3A antibodies, as reported previously^28^.

### Immunocytochemistry

The (pcDNA3.1-rabphilin-3A-V5-His) vector containing the full-length human rabphilin-3A gene was used. The vector was transfected into COS-7 cells using Lipofectamine® 2000 Reagent (Thermo Fisher Scientific). A negative control vector was not transfected into COS-7 cells. After a 24-h incubation, the cells were fixed with 4% paraformaldehyde for 20 min at room temperature. After being washed with PBS, the cells were incubated with 0.3% Triton X-100 followed by incubation with both patient serum (1:50) and anti-V5 antibodies (Invitrogen, 1:200). To examine the recognition of rabphilin-3A by patient serum, cells were stained with Alexa Fluor 488–conjugated anti-human IgG and Alexa Fluor 594–conjugated anti-mouse IgG. Colocalization was observed with a fluorescence microscope (BZ-X800; Keyence).

## Results

Fifteen consecutive patients who were diagnosed with CDI were recruited. The 15 patients consisted of 9 males and 6 females with a median age of 45.0 years (range 23–69 years) (Table 1).

**Table 1:** Clinical characteristics, MRI findings of the pituitary gland, pituitary stalk or sellar mass, the stalk diameter, and diagnosis of the underlying disease in 15 patients with central diabetes insipidus.

Case	Age/sex	Anterior pituitary dysfunction	Onset of adrenal insufficiency	Hyper-prolactinemia	MRI findings of pituitary and stalk or sellar/suprasellar mass	Diameter of the stalk (mm)	Pathology	Diagnosis
1	23/M	−	−	−	Enlargement of the pituitary gland with cystic component and stalk thickening	4.5	−	Sarcoidosis
2	68/F	GH, Gn	−	+	Enlargement of the pituitary gland with cystic component and stalk thickening	3.3	+ (resection)	Rathke cleft cyst
3	65/M	ACTH, GH, TSH, Gn	5 months after DI	−	Enlargement of the pituitary gland and stalk thickening	2.7	−	LPH
4	26/M	−	−	−	Normal size	2	−	Sarcoidosis
5	27/F	−	−	+ *	Enlargement of the pituitary gland and stalk thickening	3.1	−	LINH
6	58/M	Gn	−	−	Stalk thickening	6.3	+ (biopsy)	LPH
7	69/F	−	−	−	Stalk thickening	3.4	−	LINH
8	57/F	ACTH, Gn	Simultaneously with DI	+	Cystic lesion with heterogeneous inner component	13.8	+ (resection)	Craniopharyngioma
9	47/F	−	−	−	Stalk thickening	2.9	−	LINH
10	55/F	ACTH, GH, TSH, Gn	2 months after DI	+	Enlargement of the pituitary gland and stalk thickening	7	+ (biopsy)	LPH
11	35/M	−	−	−	Stalk thickening	3.1	−	LINH
12	67/M	ACTH, GH, TSH, Gn	Masked DI	+	Enlargement of the pituitary gland and stalk thickening	6.9	+ (biopsy)	LPH
13	48/M	ACTH, GH, TSH, Gn	6 months after DI	−	Well-defined tumor of 17 mm	15.7	+ (resection)	Germinoma
14	37/M	−	−	−	Stalk thickening	2.8	−	LINH
15	28/M	−	−	−	Cystic lesion	2.4	+ (resection)	Rathke cleft cyst

M, male; F, female; Gn, gonadotropin (LH and/or FSH); + , presence (available or positive); −, absence (not available, or negative); Hyperprolactinemia (* Case 5 was pregnant); Pathology, histopathological examination of the pituitary lesion; (biopsy), surgical biopsy of the pituitary lesion; (resection), surgical resection of the pituitary lesion; LINH, lymphocytic infundibuloneurohypophysitis; LPH, lymphocytic panhypophysitis. The diameter of the stalk was measured on sagittal MRI sections.

### Clinical characteristics and diagnosis of CDI in 15 patients

**Figure 1:**
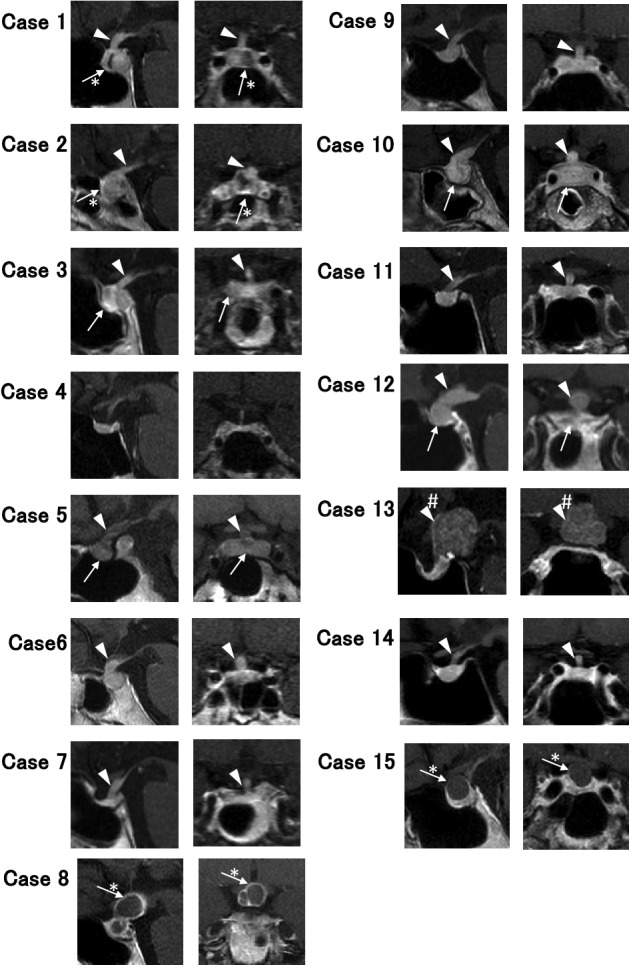
T1-weighted MR images of the pituitary in the 15 patients. The images from Case 5 were not enhanced because the patient was pregnant. All other images were enhanced by gadolinium contrast medium. Sagittal (left) and the coronal (right) sections from each patient are shown. Arrowheads indicate thickened pituitary stalks, and arrows indicate enlarged pituitary glands. *Indicates a cystic component, and #Indicates a well-defined tumor.

Fifteen patients were diagnosed with CDI according to their AVP response on the hypertonic saline infusion test, as shown in Supplementary Fig. 1. MRI scans of the pituitary gland and pituitary stalk or sellar mass are demonstrated in Fig. 1, and the MRI findings and stalk diameters are described in Table 1. The diagnoses of the underlying diseases were as follows: LINH in five cases (Cases 5, 7, 9, 11 and 14), LPH in four cases (3, 6, 10 and 12), sarcoidosis in two patients (1 and 4), Rathke cleft cyst in two cases (2 and 15), craniopharyngioma in one case (8), and intracranial germinoma in one case (13) (Table 1). Five patients (3, 8, 10, 12 and 13) suffered from anterior pituitary dysfunction, and five patients (2, 5, 8, 10 and 12), including one pregnant woman, had accompanying hyperprolactinemia (Table 1). The symptoms of anterior pituitary dysfunction were various and sometimes uncertain. We especially focused on the symptoms and signs of adrenal insufficiency, such as general malaise, appetite loss and body weight loss. The onset of adrenal insufficiency was considered to represent the onset of anterior pituitary dysfunction. CDI was followed by adrenal insufficiency in three patients (3, 10 and 13), CDI and adrenal insufficiency developed simultaneously in one patient (8), and DI was masked in one patient (12) (Table 1).

Four patients (2, 8, 13 and 15) suspected of having pituitary tumors underwent surgical resection. Three patients (6, 10 and 12) with a thickened pituitary stalk and infundibulum on MRI, in which the presence of a tumor could not be ruled out, underwent biopsy (Fig. 1). Surgical resection or biopsy was performed before treatment with therapeutic doses of glucocorticoid treatment. Following the clinical guidelines of the Japan Endocrine Society^29^, pituitary biopsy was considered when a precise diagnosis was required (e.g., suspicion of malignant tumors), or the pituitary lesion progressed during follow-up. The diagnoses were confirmed by histopathological findings.

We considered that the other eight patients did not require biopsy immediately and were instead followed up carefully (Table 1). Two patients (1 and 4), who had bilateral hilar lymphadenopathy were examined by ^67^Ga scintigraphy and lung biopsy and were diagnosed with sarcoidosis according to histopathological findings in the lung specimen. However, pituitary biopsy was not performed. Five patients (5, 7, 9, 11 and 14) were diagnosed with LINH according to clinical manifestations without histopathological examination because they had preserved anterior pituitary function and no positive findings of other diseases, including sarcoidosis, IgG4-related hypophysitis and Langerhans cell histiocytosis. No patient showed an elevated serum IgG4 level or involvement of other organs, such as the pancreas, bile duct and salivary gland. Case 3 was initially diagnosed with LINH because anterior pituitary function was preserved. He was treated with desmopressin alone, but 3 months later, his anterior pituitary function was impaired with exacerbated pituitary swelling and pituitary stalk thickening. His anterior pituitary function was reflected by the following levels: < 2.0 pg/mL ACTH, 1.4 μg/dL cortisol, 0.122 μIU/mL TSH, 2.88 pg/mL free T3, 0.67 ng/dL free T4, 0.52 ng/mL GH, 65 ng/mL IGF-I, 0.23 mIU/mL LH, 1.27 mIU/mL FSH, and 4.8 ng/mL testosterone. He was eventually diagnosed with LPH according to his clinical course. The eight patients who were diagnosed clinically without histopathological examination were followed up carefully over a range of 1 year 5 months to 7 years 9 months (Table 2).

**Table 2:** Clinical/histopathological diagnosis, presence of anti-rabphilin-3A (RPH3A) antibodies, and the interval between onset and blood sampling in 15 patients with central diabetes insipidus.

Case	Diagnosis	RPH3A antibodies	Interval between the onset and blood sampling	Follow-up periods after clinical diagnosis
5	LINH	+	1 month	5 years 5 months
7	LINH	+	1 month	4 years 6 months
9	LINH	+	2 months	3 years 9 months
11	LINH	+	2 months	2 year 4 months
14	LINH	−	3 months	1 year 5 months
3	LPH	+	2 months	6 years 10 months
6	LPH	+	2 months	−
10	LPH	−	6 months	−
12	LPH	+	1 month	−
1	Sarcoidosis	+	1 month	7 years 9 months
4	Sarcoidosis	−	6 months	6 years 2 months
2	Rathke cleft cyst	−	2 months	−
15	Rathke cleft cyst	−	1 month	−
8	Craniopharyngioma	−	4 months	−
13	Germinoma	+	7 months	−

The follow-up period (last diagnosis) is provided for the patients who were diagnosed clinically without histopathological examination.

The following complications were observed. In Case 5, CDI onset occurred during the third trimester of pregnancy; the details have been reported previously^35^. Case 6 was treated for type 2 diabetes mellitus with oral hypoglycemic drugs previously. However, the diagnosis was corrected to slowly progressive type 1 diabetes mellitus because of a high titer of anti-glutamic acid decarboxylase antibodies. In addition, he was diagnosed with Graves’ disease with positivity for anti-TSH receptor antibodies, as well as seropositivity for anti-gastric parietal cell antibodies. Case 7 developed Graves’ disease 5 months after the onset of CDI. The complication of Graves’ disease supports the diagnosis of lymphocytic hypophysitis.

### Histopathology

**Figure 2:**
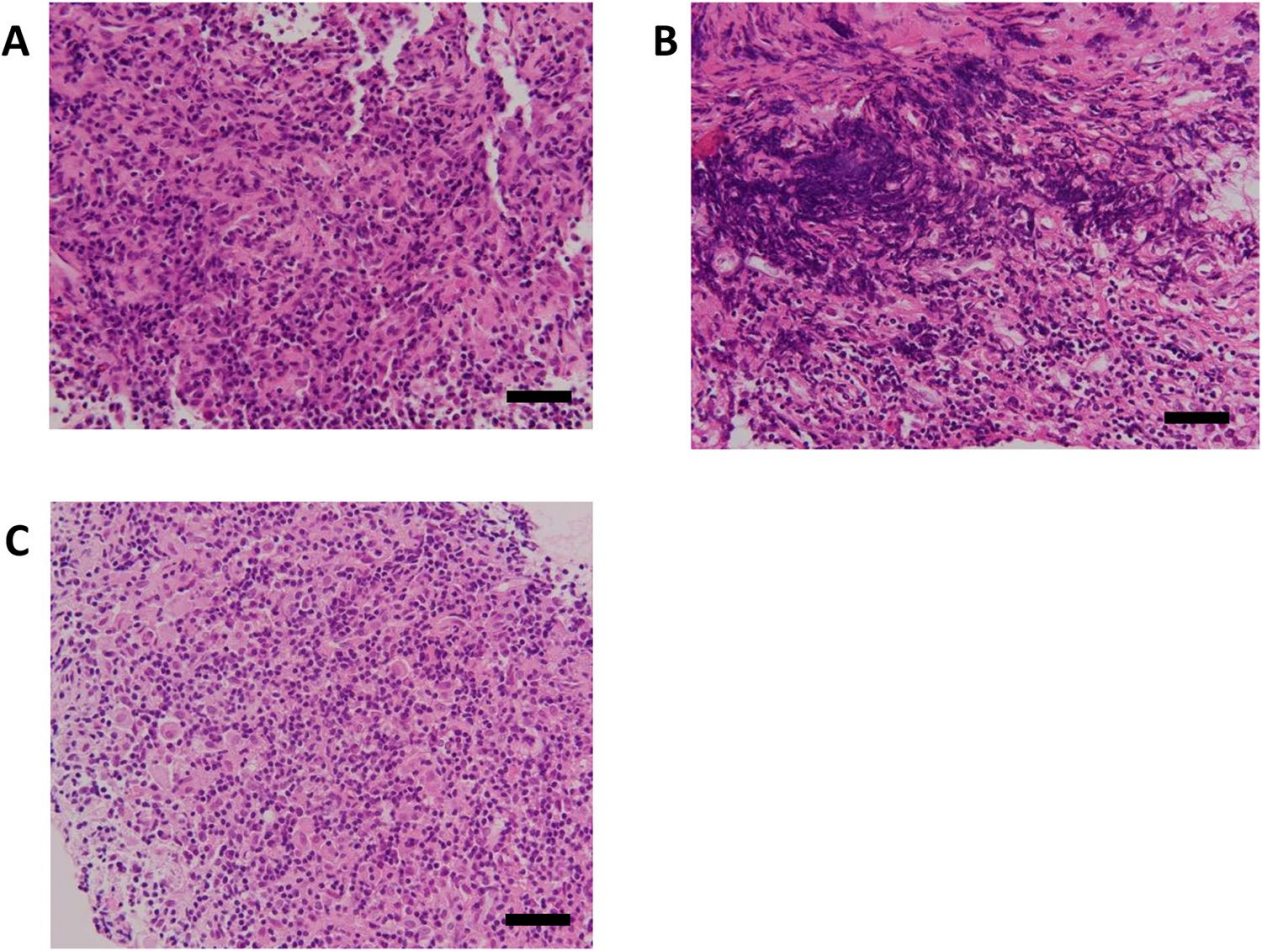
Microphotographs from Cases 6, 10 and 12 of the pituitary tissues stained with hematoxylin and eosin. (**A**) Microphotographs from Case 6 demonstrating lymphocytic infiltration in the posterior pituitary. (**B**) Microphotograph from Case 10 demonstrating fibrotic and lymphocytic infiltration in pituitary tissue. (**C**) Microphotograph from Case 12 demonstrating infiltration of large histiocytes and small lymphocytes in pituitary tissue. Scale bars indicate 50 μm.

Cases 2 and 15 were diagnosed as Rathke cleft cyst and Case 8 was as craniopharingioma, by histopathological studies (data not shown). Cases 6, 10, and 12 were diagnosed as LPH by histopathological studies, and microphotographs of hematoxylin- and eosin-stained pituitary tissue sections from these cases are shown in Fig. 2. In Case 6 (Fig. 2A), lymphocytes and plasma cells had infiltrated the posterior pituitary with mild fibrotic change, and small lymphocytes had infiltrated the anterior pituitary. IgG4-positive cells were observed only in a limited region. Therefore, we considered that IgG4 was not a major cause of the hypophysitis, and that the diagnosis was compatible with LPH. In Case 10 (Fig. 2B), lymphocytic infiltration was observed in the anterior pituitary with trabecular structure destruction as well as in the posterior pituitary with reactive hyperplasia. These histopathological findings were consistent with LPH. In Case 12 (Fig. 2C), infiltration of large cells was observed, and immunohistochemistry revealed that these cells were positive for CD68, and negative for CD1a. However, findings of sarcoidosis or Langerhans cell histiocytosis were not detected in any specimen. These histopathological findings were consistent with LPH (data not shown).

**Figure 3:**
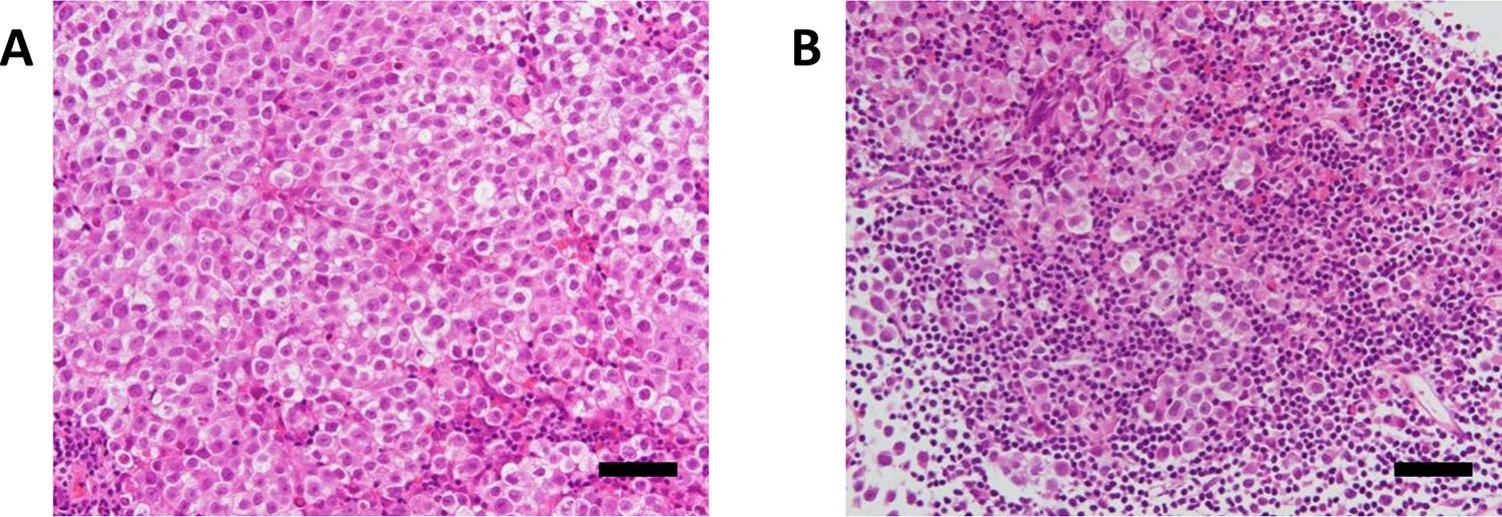
Microphotographs of germinoma tissues stained with hematoxylin and eosin from Case 13. (**A**) Microphotograph of the region in which tumor cells were predominant. (**B**) Microphotograph of the region in which lymphoplasmacytic infiltration was predominant. Scale bars indicate 50 μm.

Case 13 was diagnosed as pure germinoma because no teratoma component was observed. The tumor tissue comprised round, large tumor cells and lymphocytes (Fig. 3). The round, large tumor cells were predominant in some regions of the tumor tissue (Fig. 3A), whereas infiltration of lymphocytes and plasma cells was observed predominantly in the other region of the tumor tissue (Fig. 3B).

### Anti-rabphilin-3A antibodies

**Figure 4:**
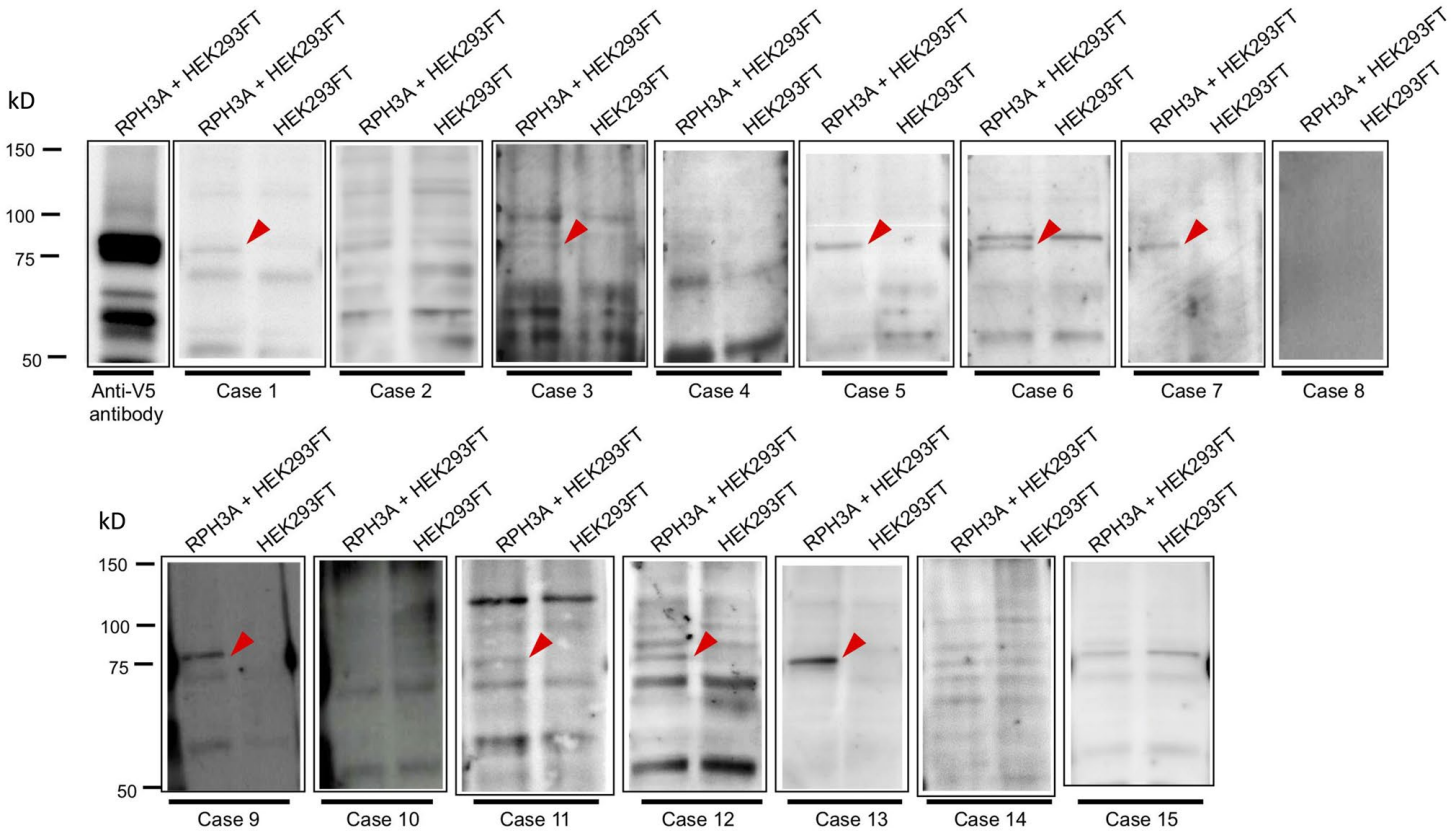
Detection of anti-rabphilin-3A antibodies by Western blotting. Recombinant full-length human rabphilin-3A expressed in HEK293FT cells (RPH3A + HEK293FT, left lane in each case) or negative control (HEK293FT, right lane in each case) were probed with serum from Case 1 to 15. The arrowhead indicates the presence of anti-rabphilin-3A antibodies in serum. Recombinant full-length human rabphilin-3A expressed in HEK293FT cells was also probed with an anti-V5 antibody as positive control (Anti-V5 antibody) in the first lane from the left. Cropped Western blots were from different gels and were made explicit using delineation with dividing lines.

The interval between the onset of CDI or anterior pituitary dysfunction and blood sampling to measure anti-rabphilin-3A antibodies ranged from 1 to 7 months (Table 2). Anti-rabphilin-3A antibodies evaluated by western blotting were positive in nine patients: four of the five with LINH, three of four with LPH, one of two with sarcoidosis and the one patient with germinoma (Fig. 4, Table 2). Among the seven patients definitively diagnosed by pituitary lesion biopsy, anti-rabphilin-3A antibodies were positive in two of the three with LPH (Cases 6 and 12) and in the one with intracranial germinoma (Case 13) (Table 2). The full blotting images are shown in Supplementary Figs. 2–16.

We further performed confirmatory experiments of immunocytochemistry using the seven most recently obtained samples (Cases 9–15) (Supplementary Figs. 17–23) and five healthy subjects (Supplementary Figs. 24–28). The results of the western blotting analysis regarding anti-rabphilin-3A antibodies were confirmed by immunocytochemistry; *i.e*., anti-rabphilin-3A antibodies by immunocytochemistry were positive in Case 9 (LINH), negative in Case 10 (LPH), positive in Case 11 (LINH), positive in Case 12 (LPH), positive in Case 13 (germinoma), negative in Case 14 (LINH), and negative in Case 15 (Rathke cleft cyst). Anti-rabphilin-3A antibodies by immunocytochemistry were negative in all healthy subjects.

### Treatment after the diagnosis

Immediately after the diagnosis of CDI, all 15 patients were treated with desmopressin, and Cases 8, 10, 12 and 13 were additionally treated with corticosteroids and Cases 10, 12 and 13 further treated with levothyroxine to compensate for hormone insufficiencies. None of the patients with GH deficiency (2, 10, 12 and 13) were treated with GH replacement because Cases 2 and 12 had concomitant diabetes mellitus, Case 10 refused GH replacement therapy, and Cases 13 underwent careful observation for tumor recurrence after chemoradiotherapy for his germinoma. Case 8 suffered from GH deficiency after removal of her craniopharyngioma, but GH replacement was suspended in lieu of careful observation for tumor recurrence. Of the six patients with hypogonadotropic hypogonadism, only Case 13 was treated with sex hormone replacement therapy, because the other five patients (2, 6, 8, 10 and 12) were older and in menopause or refused sex hormone replacement therapy.

The four patients with LPH (3, 6, 10 and 12) were treated with glucocorticoids as shown below. Case 3 was treated with 30 mg/day prednisolone for 14 days and then tapered off. The swelling of his pituitary improved slightly, and GH and gonadotropin levels were restored, but treatment with both corticosteroids and levothyroxine was continued. Case 6 had concomitant CDI and hypogonadotropic hypogonadism and was diagnosed with LPH. He was treated with steroid pulse therapy, after which his anterior pituitary function recovered, as indicated by increases in the LH, FSH and testosterone levels from 0.16 to 2.30 mIU/mL, 0.51 to 4.29 mIU/mL and 9.4 to 573.8 ng/mL, respectively. Cases 10 and 12 displayed panhypopituitarism and thus were treated with steroid pulse therapy, after which anterior pituitary function recovered slightly, but the treatment with corticosteroids and levothyroxine was indispensable.

Of the two patients of sarcoidosis, Case 1 was treated with prednisolone (30 mg/day) for 14 days, and then tapered off. Case 4 was not treated with glucocorticoids because his pituitary was not swollen, and no other organ involvement was noted except for bilateral hilar lymphadenopathy. The one patient (13) who was diagnosed with an intracranial germinoma was treated with chemoradiotherapy, followed by androgen replacement therapy.

## Discussion

This is the first time that anti-rabphilin-3A antibodies were studied in consecutive 15 patients with CDI. Of these 15 patients, anti-rabphilin-3A antibodies were positive in nine patients, including four of five with LINH, three of four with LPH, one of two with sarcoidosis, and the one patient with germinoma. Particularly, among the seven patients definitively diagnosed by biopsy, two of the three with LPH and the one with germinoma were positive for anti-rabphilin-3A antibodies. We also confirm the positivity of anti-rabphilin-3A antibodies in two patients with biopsy proven LPH and one patient with biopsy proven germinoma by immunocytochemical analysis. These findings suggest that anti-rabphilin-3A antibodies are relatively frequent in CDI concomitant with LINH as well as in LPH. We are the first to demonstrate the presence of anti-rabphilin-3A antibodies in patients with biopsy-proven LPH and germinoma.

CDI is caused by insufficient secretion of AVP from the posterior pituitary and by a variety of disorders arising mainly from the hypothalamus. It has been suggested that an autoimmune process is involved in the pathogenesis of CDI with hypophysitis^2,4,5,10,12,26,28,36–38^. Determining the cause of CDI is sometimes difficult without biopsy. However, the use of biopsy is limited because of its invasiveness, especially in children. Therefore, the development of non-invasive diagnostic tests or markers for differentiating among the various causes of CDI (*e.g.*, autoimmune hypophysitis and tumors such as germinoma) is very important, and such markers have been investigated. Anti-vasopressin-cell antibodies have been detected in patients with idiopathic CDI; however, these antibodies have also been detected in DI of other etiologies, including Langerhans cell histiocytosis and germinoma, and thus cannot be considered a reliable marker of autoimmune mediated CDI^26,27^.

Anti-rabphilin-3A antibodies were shown to be a diagnostic marker of LINH with high sensitivity and specificity^28^. In addition, a case of anti-rabphilin-3A antibody-positive CDI that developed during pregnancy has been reported^34,39^. Furthermore, a case of childhood-onset LINH in a 10-year-old boy who was identified as positive for anti-rabphilin-3A antibodies at 9 years after CDI onset was reported recently^40^. In the present study, 9 of the 15 patients of CDI were positive for anti-rabphilin-3A antibodies. The anti-rabphilin-3A antibody positivity in four of the five patients of LINH is similar to previously reported prevalence of 18 of 25 (72%) patients with clinically diagnosed LINH and 4 of 4 (100%) patients with biopsy-proven LINH. Therefore, the findings of the present study support that anti-rabphilin-3A antibody is a diagnostic marker for LINH with good sensitivity.

One of the most important findings of the present study was anti-rabphilin-3A antibody positivity in 2 of the 3 biopsy-proven patients with LPH. This is the first report of the presence of anti-rabphilin-3A antibodies in LPH, a subtype of lymphocytic hypophysitis that affects both of the anterior and posterior pituitary gland and causes CDI. Three patients (6, 10 and 12) were diagnosed with LPH by histopathological examination, of whom two (6 and 12) were positive for anti-rabphilin-3A antibodies. Although little is known about the etiology of LPH, an autoimmune mechanism is thought to be involved in the pathophysiology of LPH. Our data suggest that an abnormal immune response against rabphilin-3A may be involved in CDI concomitant with LPH. A previous study reported that patients with CDI were positive for anti-hypothalamus antibodies (AHAs) to AVP-secreting cells, and that those patients were positive for AHAs to CRH-secreting cells and/or anti-pituitary antibodies to ACTH/GH-secreting cells^41^. These finding suggest that the autoimmune impairment of the hypothalamus and/or pituitary, rather than expansion of the inflammatory pituitary process to the posterior pituitary and infundibulum, may be responsible for CDI and/or may contribute to hypopituitarism. It is still unknown whether the severe inflammation in the posterior pituitary extends to the adjacent anterior pituitary, and therefore LPH is diagnosed by pathological or endocrinological findings. In this regard, it is noteworthy that Case 3 in the present study was similar to a previously reported case^42^, in that both patients initially suffered from CDI and were positive for anti-rabphilin-3A antibodies, followed by the development of anterior pituitary dysfunction. It is possible that the swelling of the posterior pituitary and pituitary stalk compressed the anterior pituitary and caused anterior pituitary dysfunction; then, anterior pituitary dysfunction was partially, but not fully, restored after improvement of the swelling. These cases suggest that autoimmune impairment of the hypothalamus and/or pituitary may cause CDI and anterior pituitary dysfunction. In addition, considering that the expression of rabphilin-3A is not detected in the anterior lobe^28^, our Case 3 and the previously reported case may provide clues regarding the origin or pathogenesis of LPH.

Interestingly, one patient of pathologically proven germinoma (Case 13) was positive for anti-rabphilin-3A antibodies, representing the first case of biopsy-proven germinoma with anti-rabphilin-3A antibodies positivity. In a previous report, anti-rabphilin-3A antibodies were negative in all the 34 biopsy-proven samples from sellar/suprasellar masses, including 5 germinomas, yielding a specificity of 100% for distinguishing sellar/suprasellar masses^28^. The reason for the presence of anti-rabphilin-3A antibodies in Case 13 is unclear. However, pituitary and systemic antibodies were detected in a patient with an intrasellar germinoma^43^, and it was recently reported that the levels of anti-pituitary antibodies or AHAs was significantly increased in patients with germinoma^44,45^. Brain tumors, especially germinomas, might be associated with the development of hypothalamic–pituitary antibodies and pituitary defects^44,45^. In addition, Takemi et al. reported that patients with germinomas with a lower in tumor cell content (higher immune cell infiltration) had a significantly longer progression‐free survival compared with those with a higher tumor cell content, suggesting that infiltrating immune cells play an important role in predicting the treatment response^46^. Notably, infiltration of lymphocytes and plasma cells was observed predominantly in a certain region of the tumor tissue in Case 13 (Fig. 3B). It is therefore tempting to speculate that lymphocytes and plasma cells infiltrated the posterior pituitary and caused generation of anti-rabphilin-3A antibodies in Case 13. Taken together, germinoma tumor cells or a certain degree of inflammation in the posterior pituitary may promote generation of anti-rabphilin-3A antibodies. Further studies are needed to clarify the significance of the presence of anti-rabphilin-3A antibodies in germinomas.

Case 1 was diagnosed as sarcoidosis by pathological findings in the lung specimen, but not in the pituitary lesion. Therefore, the possibility of coinciding LINH and sarcoidosis cannot be completely ruled out. Another possibility may be that the sarcoidosis lesions extended to the posterior pituitary, where they induced inflammation and generation of anti-rabphilin-3A antibodies. Histological analysis of the pituitary is needed to conclude the significance of the presence of anti-rabphilin-3A antibodies in this case.

The present studied patient group is small and heterogenous for evaluation of prevalence of anti-rabphilin-3A antibodies in the patients with CDI. Since lymphocytic hypophysitis (including LINH) that causes CDI is a rare, a multicenter study is warranted to examine its prevalence.

Rabphilin-3A is a specific GTP-Rab3A-binding protein localized in secretory granules, where it regulates neurotransmitter release^47–50^. Regarding the involvement of rabphilin-3A in AVP secretion, AVP secretion was reported to be mediated by the interaction between rabphilin-3A and cullin-associated and neddylation-dissociated protein 1^51^. Rabphilin-3A may be a pathogenic antigen, and T cells specific for rabphilin-3A may be involved in the pathogenesis of neurohypophysitis in mice^52^. We previously reported that immunization of mice with rabphilin-3A led to neurohypophysitis in a mouse. Lymphocytic infiltration was observed in the neurohypophysis where rabphilin-3A was expressed but not in the anterior pituitary where rabphilin-3A was not expressed. In addition, mice immunized with rabphilin-3A showed an increase in the volume of hypotonic urine. Abatacept, which suppresses T-cell activation, ameliorated lymphocytic infiltration of CD3 + T cells in the neurohypophysis and restored the urine volume in mice immunized with rabphilin-3A^52^.

In conclusion, our current data support that anti-rabphilin-3A antibodies provide a sensitive marker of LINH in patients with CDI. In addition, we demonstrated that some patients with CDI concomitant with LPH are positive for anti-rabphilin-3A antibodies.

## Ethics approval

This study was approved by the ethics committees of Sendai Medical Center(RIN31-32), Fujita Health University (HM17-91) and Nagoya University (1355). All procedures involving human participants were performed in accordance with the ethical standards of each institutional research committee and with the 1964 Helsinki declaration and its later amendments.

## Consent to participate

Institutional review board at the Sendai Medical Center (RIN31-32) approved the use of opt-out consent. Consent was obtained in the form of opt-out in the hospital and on the web-site. We put up posters in the hospital and web-site, as follows, "We investigate the cause of central diabetes insipidus by various methods of examinations."

## Supplementary Information

Supplementary Information.
