# Supplementary material for: Studies on anti-rabphilin-3A antibodies in 15 consecutive patients presenting with central diabetes insipidus at a single referral center
Source: Sci Rep. 2022 Mar 15;12:4440. doi: 10.1038/s41598-022-08552-y (PMC8924241; doi:10.1038/s41598-022-08552-y)

Studies on anti-rabphilin-3A antibodies in 15 consecutive patients presenting with central diabetes insipidus at a single referral center

Zenei Arihara, Kanako Sakurai, Satsuki Niitsuma, Ryota Sato, Shozo Yamada, Naoko Inoshita, Naoko Iwata, Haruki Fujisawa, Takashi Watanabe, Atsushi Suzuki, Kazuhiro Takahashi, Yoshihisa Sugimura

Supplementary Information:

Supplementary figure 1: Results of the hypertonic saline infusion tests. (A) Results from the patients (Cases 1-4) who were measured using the AVP-RIA kit Neo Mitsubishi. (B) Results from the patients (Cases 5-14) who were measured using the AVP kit Yamasa.

Supplementary figures 2-16: Full-length blotting images of Figure 5 are shown in Supplementary figures 2 (anti-V5 antibody, and Cases 1), and 3-16 (Cases 2-15). Blotswith cropping line are shown in each Supplementary figure.

Supplementary figures 17-28: Immunocytological images of anti-rabphilin-3A antibodies in COS-6 cells are shown in Supplementary figures 17(Case 9), 18 (Case 10), 19 (Case 11), 20 (Case 12), 21 (Case 13), 22 (Case 14), 23 (Case 15), and 24-28 (negative control). Triple immunofluorescence shows the reactivity with serum (shown in green), V5 antibody (shown in red), and 4’6-diamidino-2-phenylindole (DAPI) antibody (shown in blue). Scale bars indicate 50 μm (white bars).

Supplementary figure 1

(A)

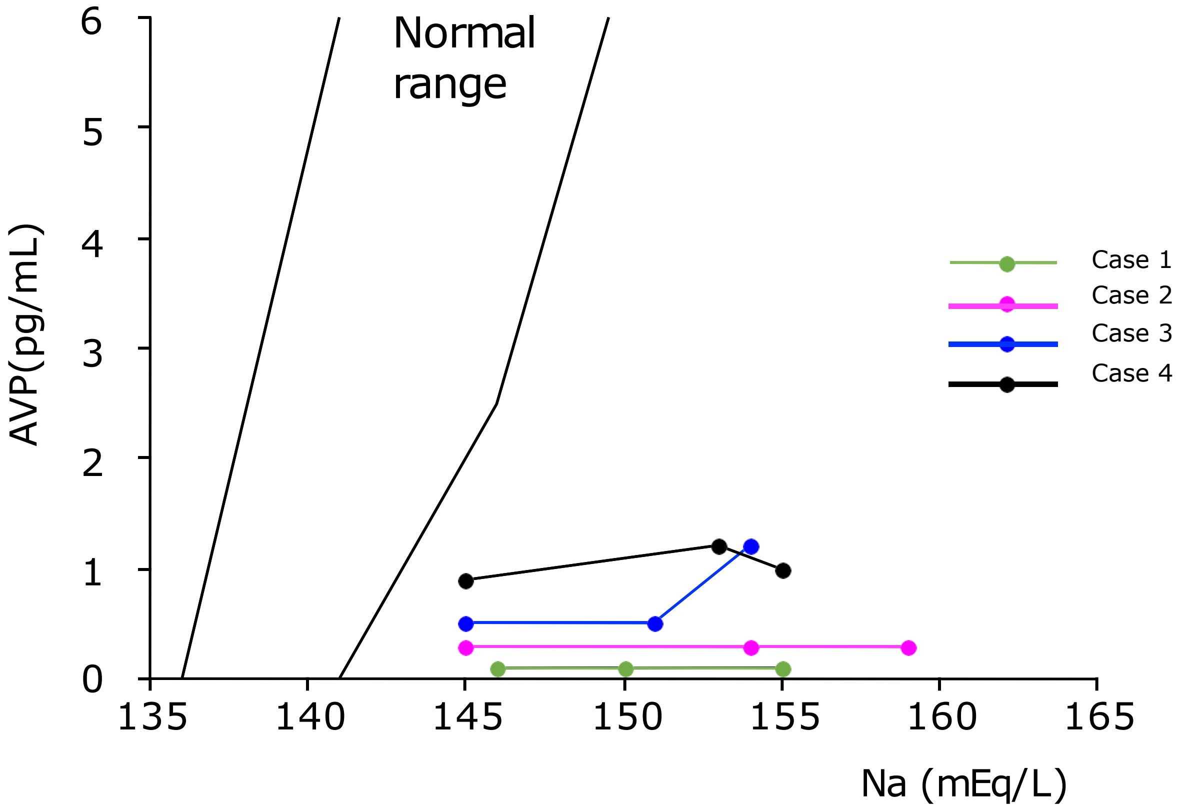

(B)

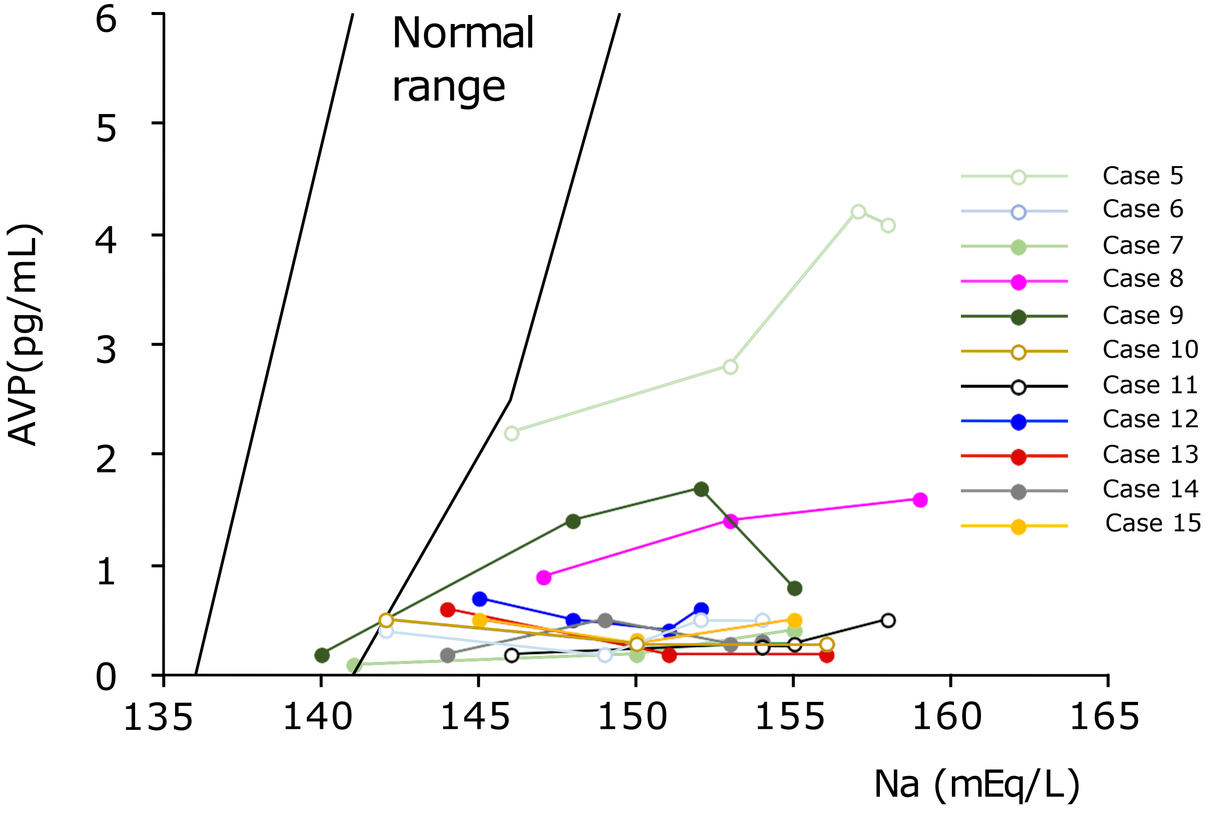

Supplementary figure 2

Original image of figure 4 (Case 1)

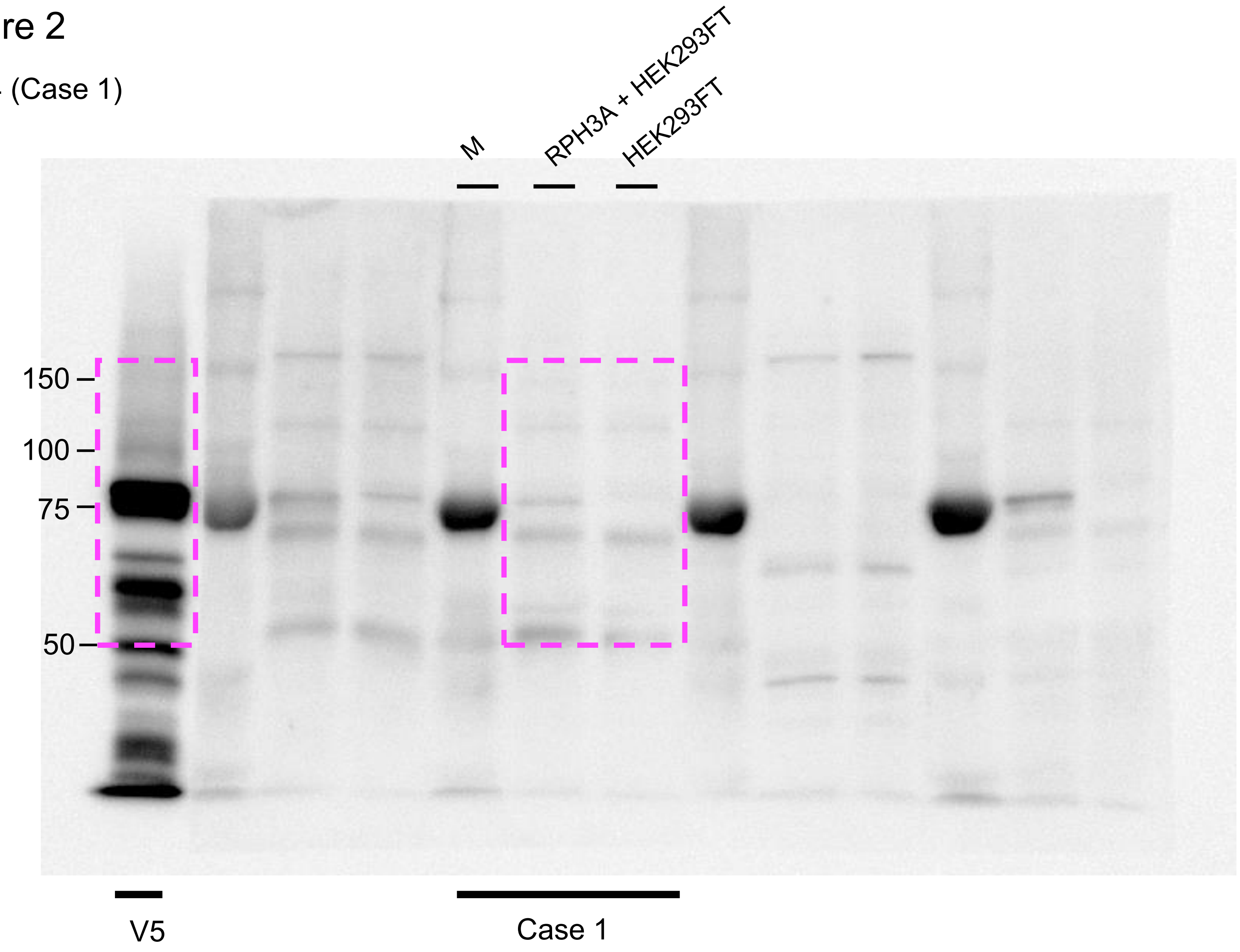

Supplementary figure 3

Original image of figure 4 (Case 2)

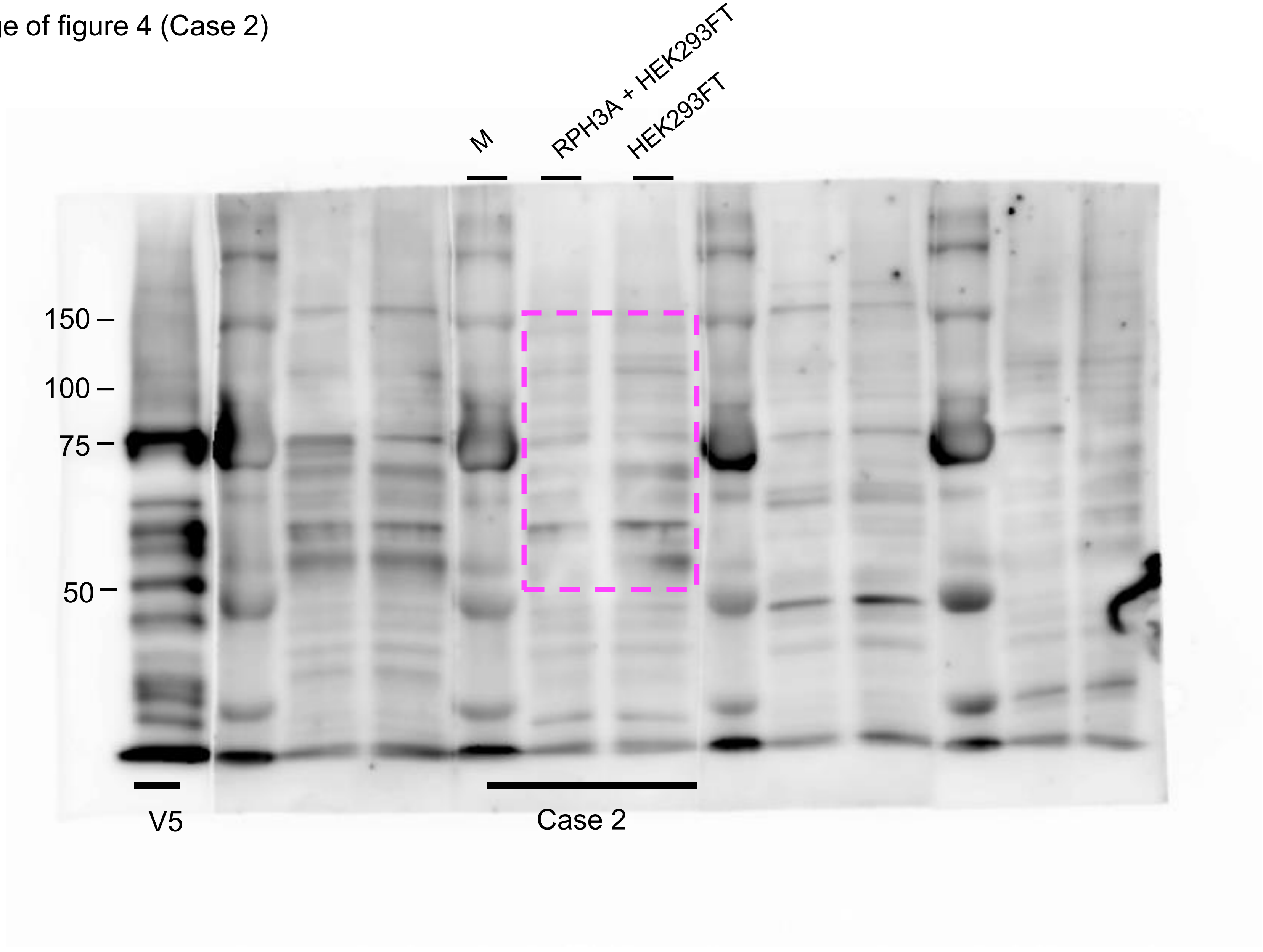

Supplementary figure 4

Original image of figure 4 (Case 3)

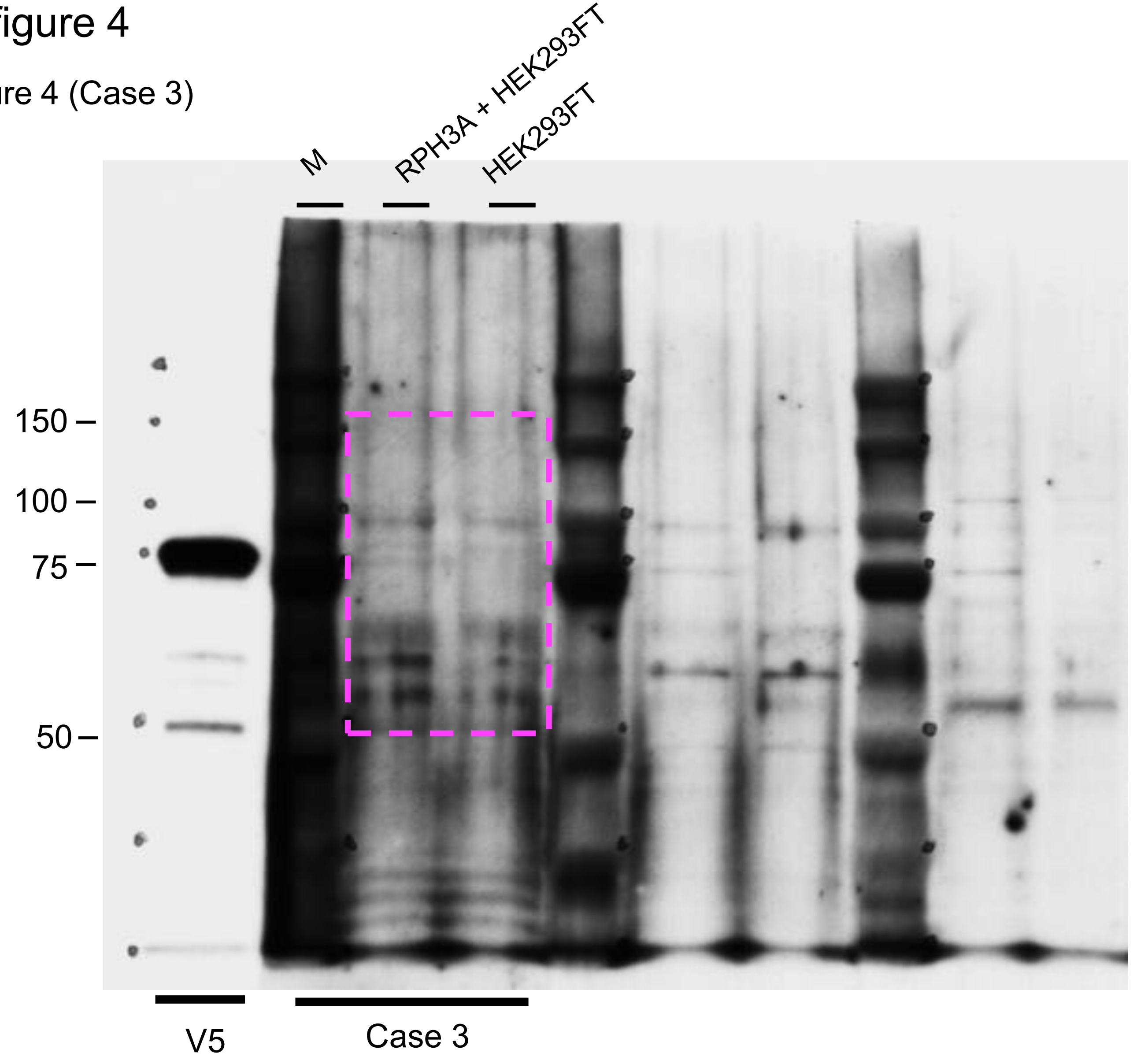

Supplementary figure 5

Original image of figure 4 (Case 4)

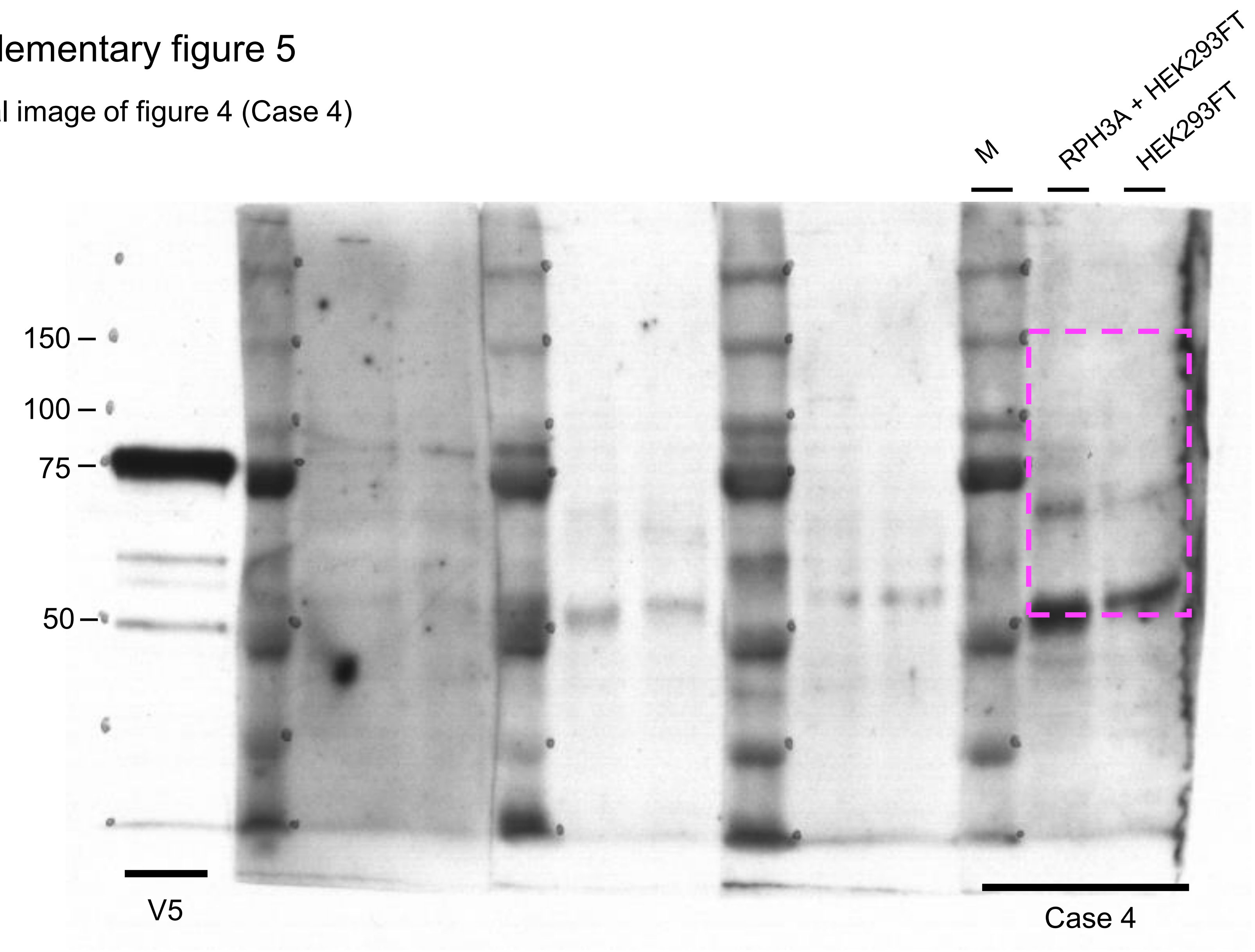

Supplementary figure 6

Original image of figure 4 (Case 5)

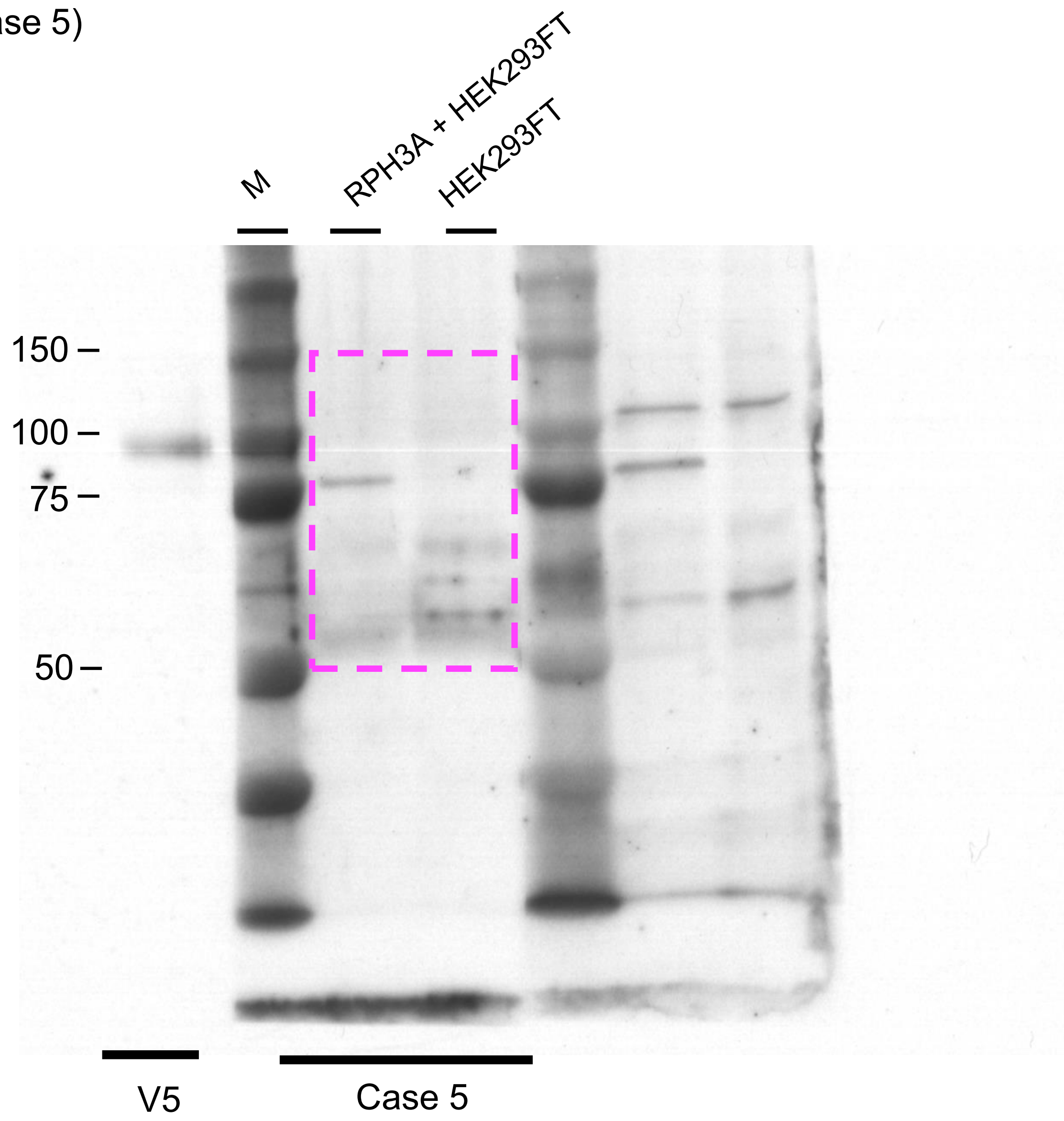

Supplementary figure 7

Original image of figure 4 (Case 6)

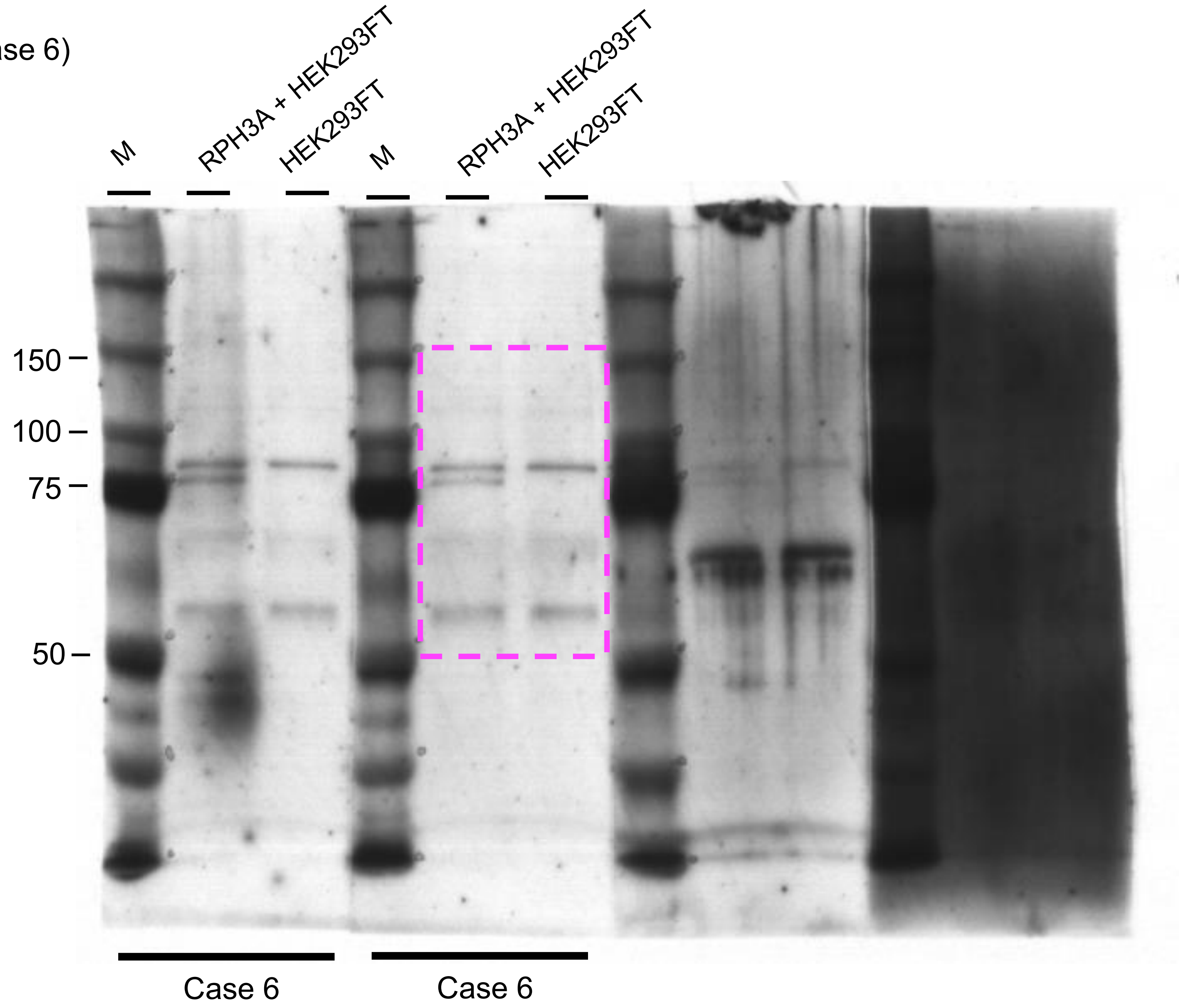

Supplementary figure 8

Original image of figure 4 (Case 7)

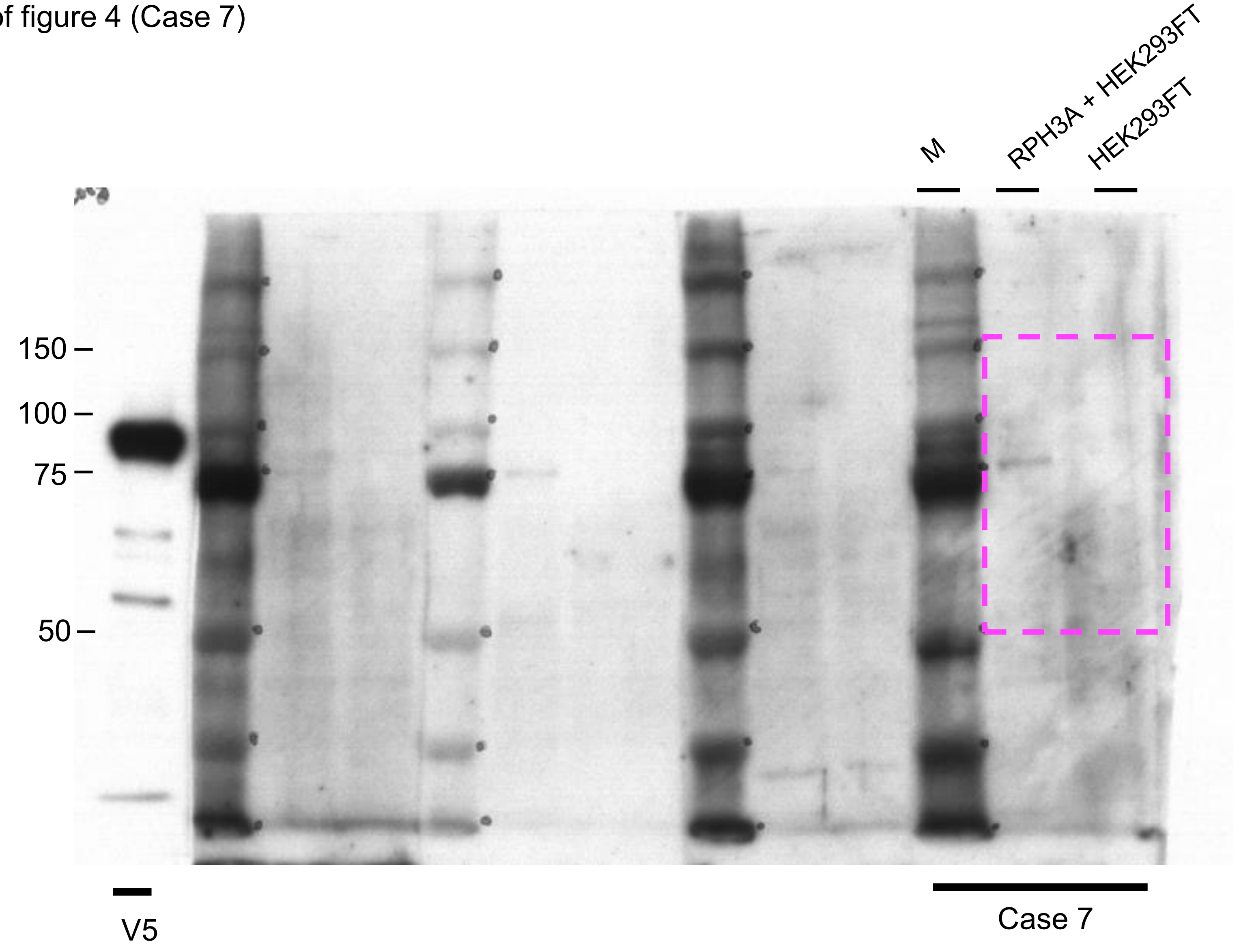

Supplementary figure 9

Original image of figure 4 (Case 8)

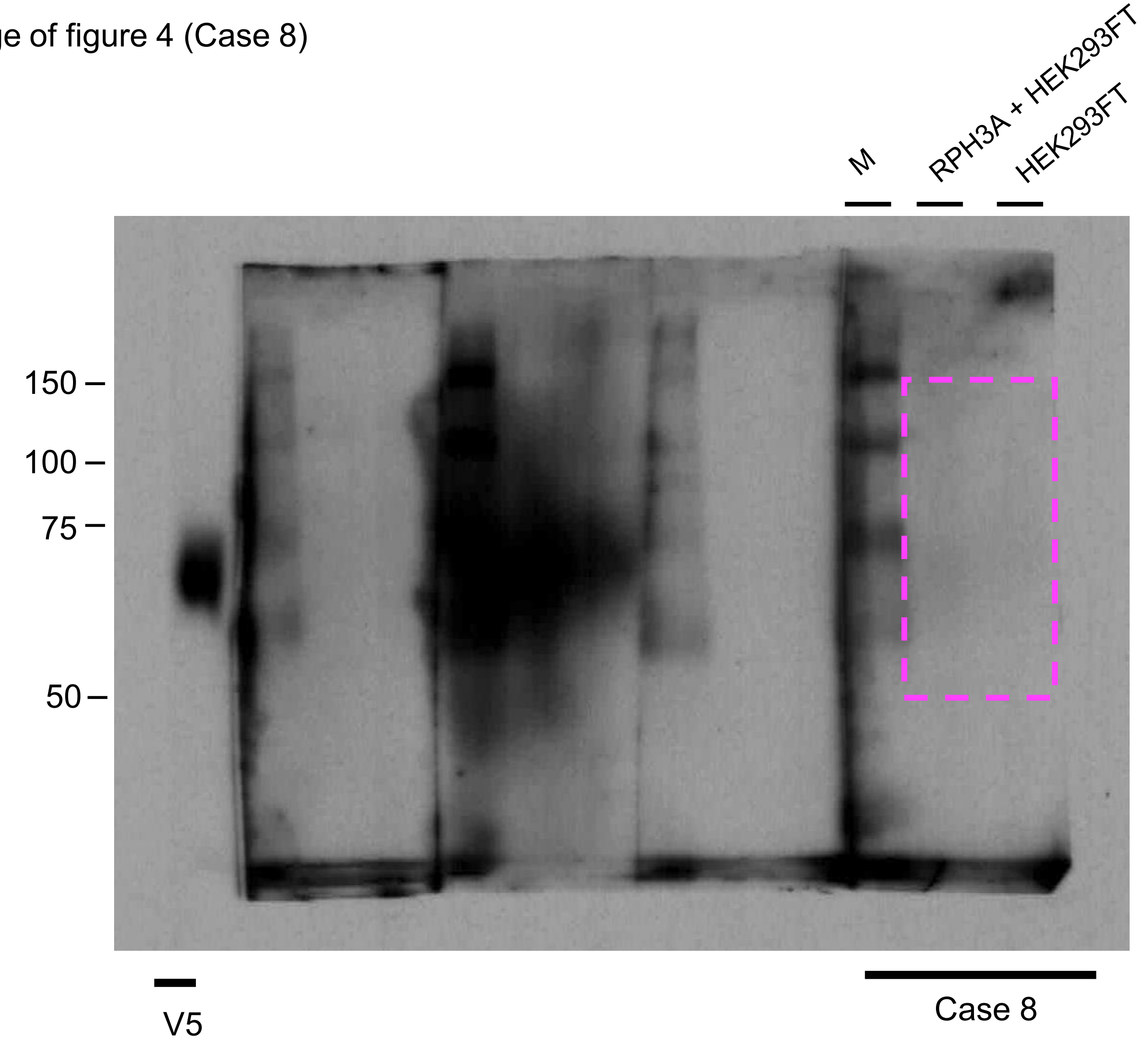

Supplementary figure 10

Original image of figure 4 (Case 9)

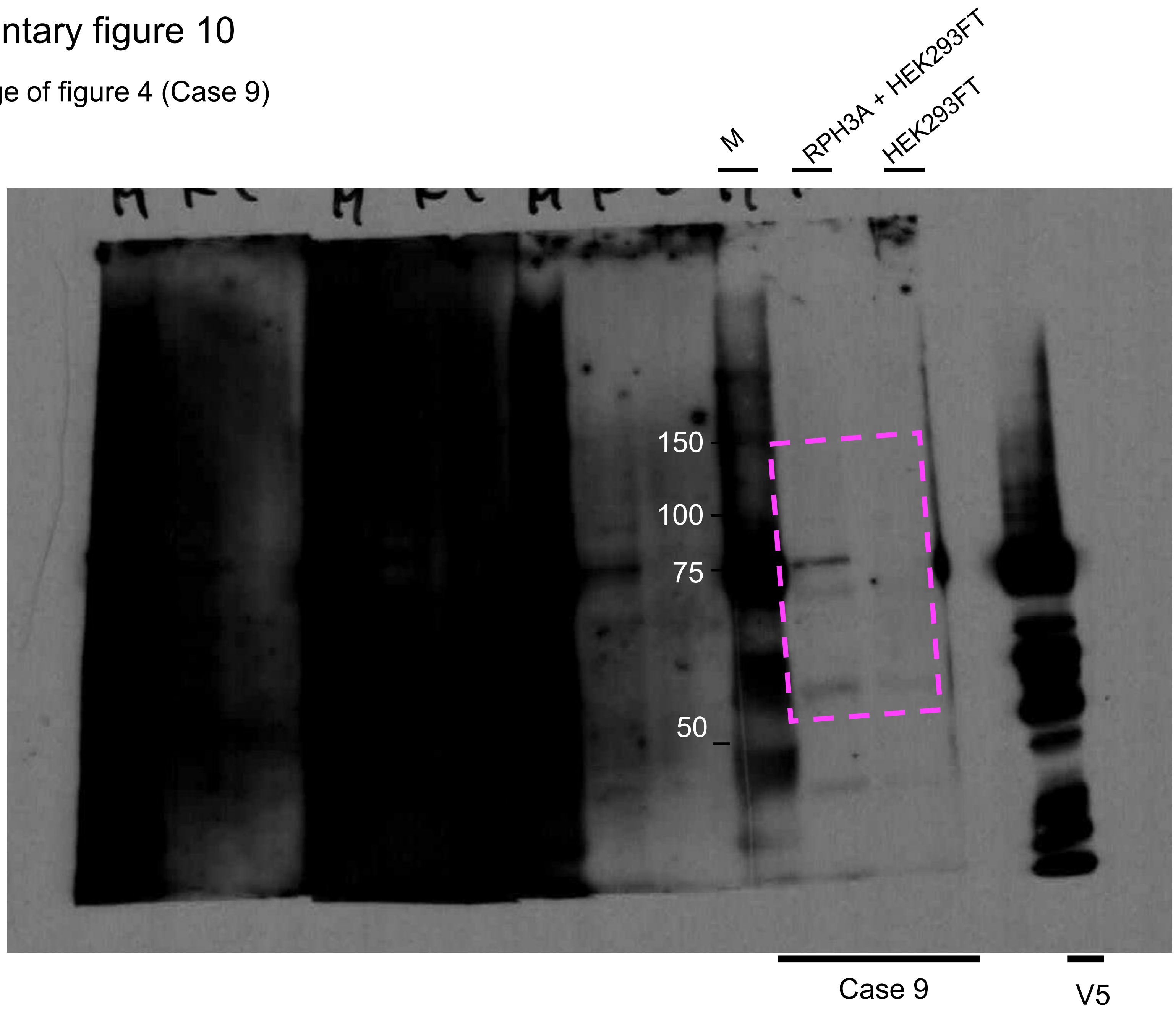

Supplementary figure 11

Original image of figure 4 (Case 10)

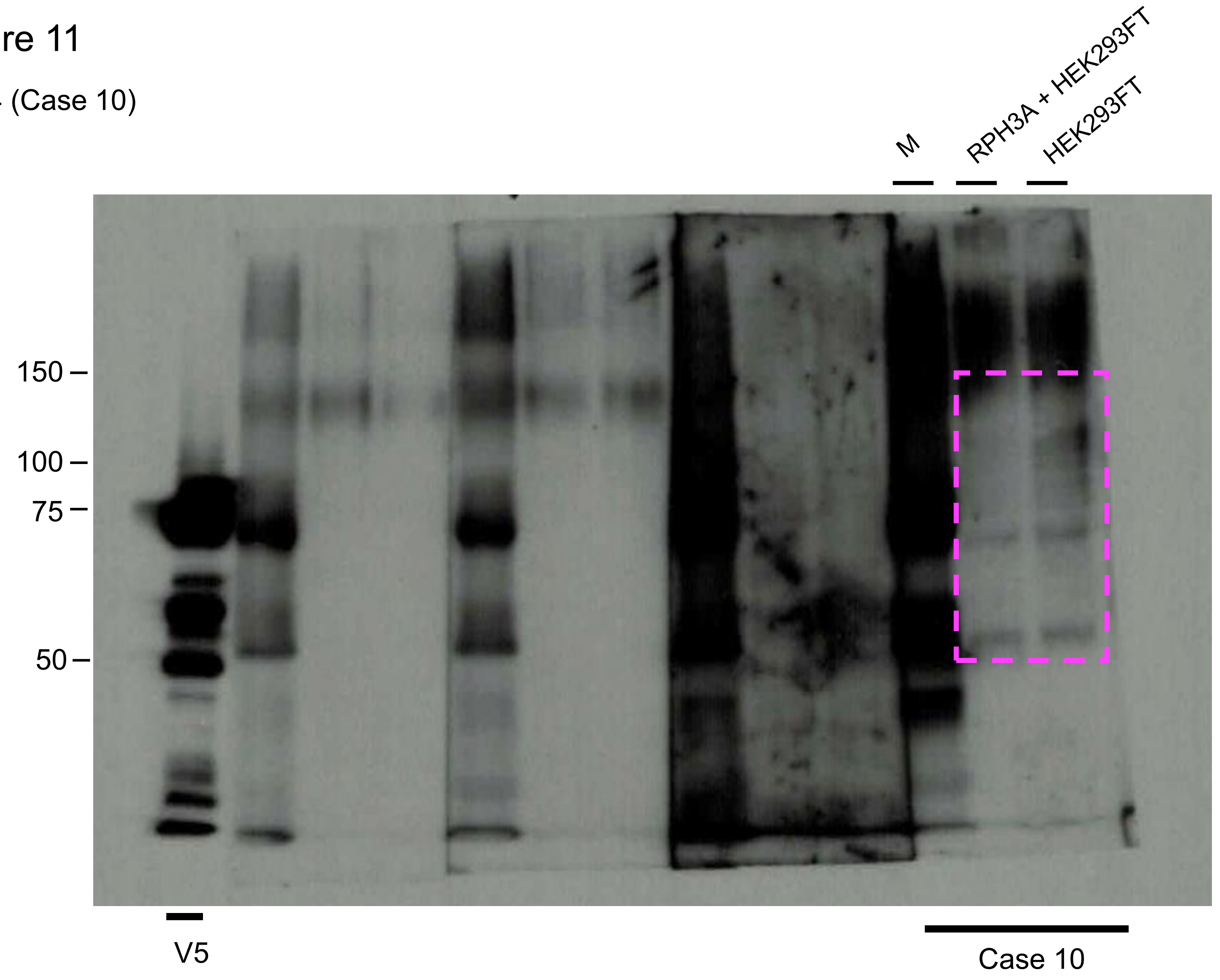

Supplementary figure 12

Original image of figure 4 (Case 11)

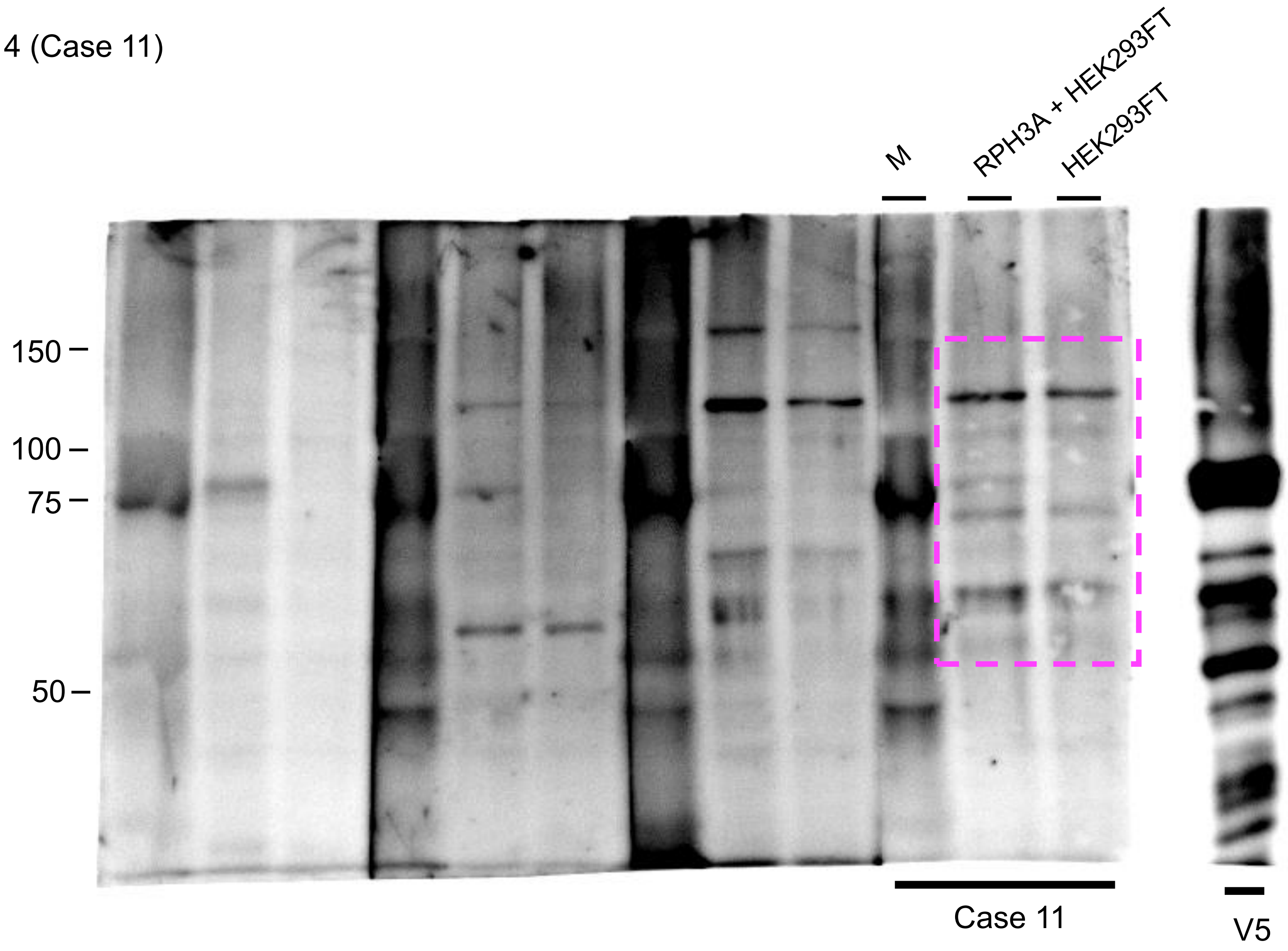

Supplementary figure 13

Original image of figure 4 (Case 12)

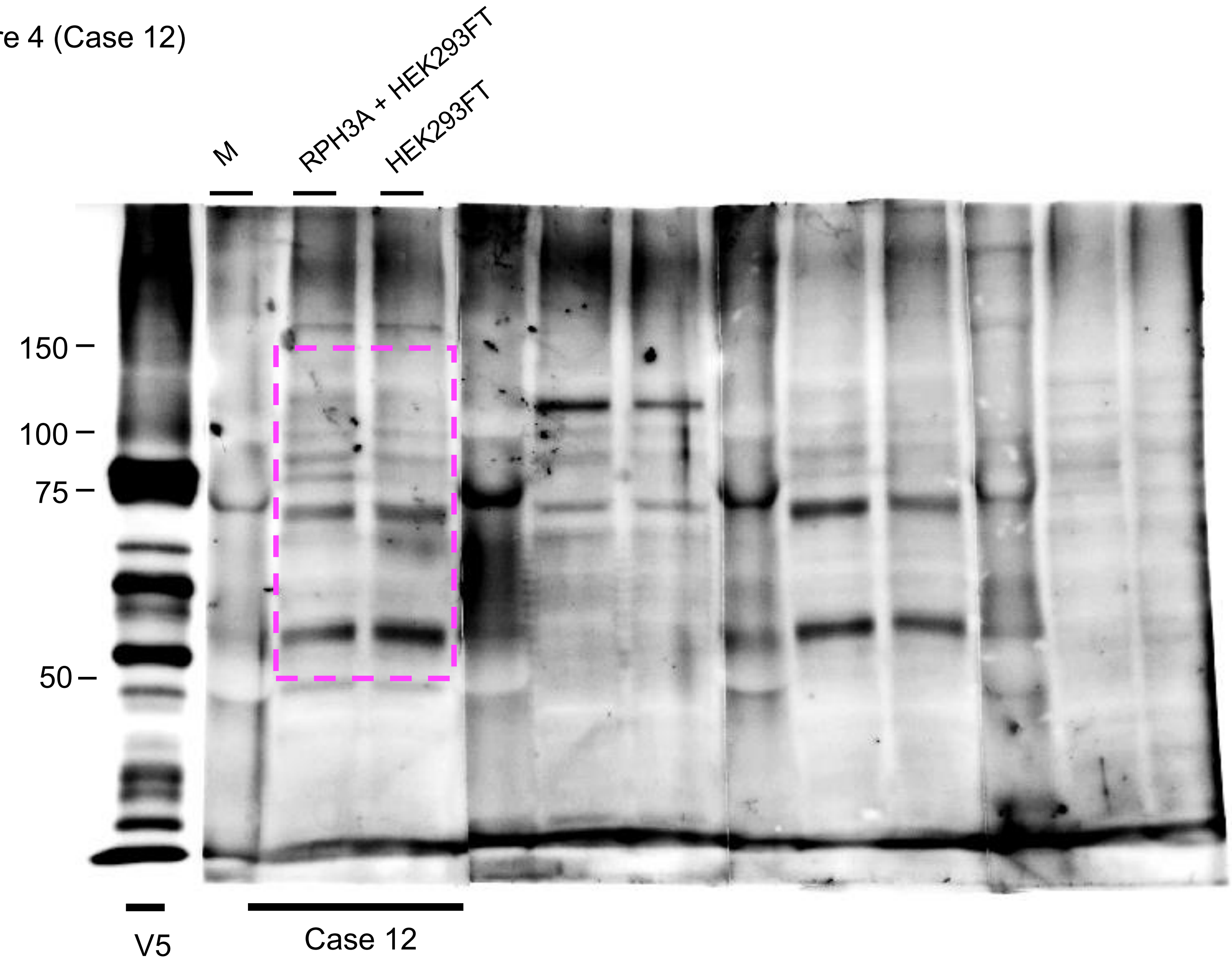

Supplementary figure 14

Original image of figure 4 (Case 13)

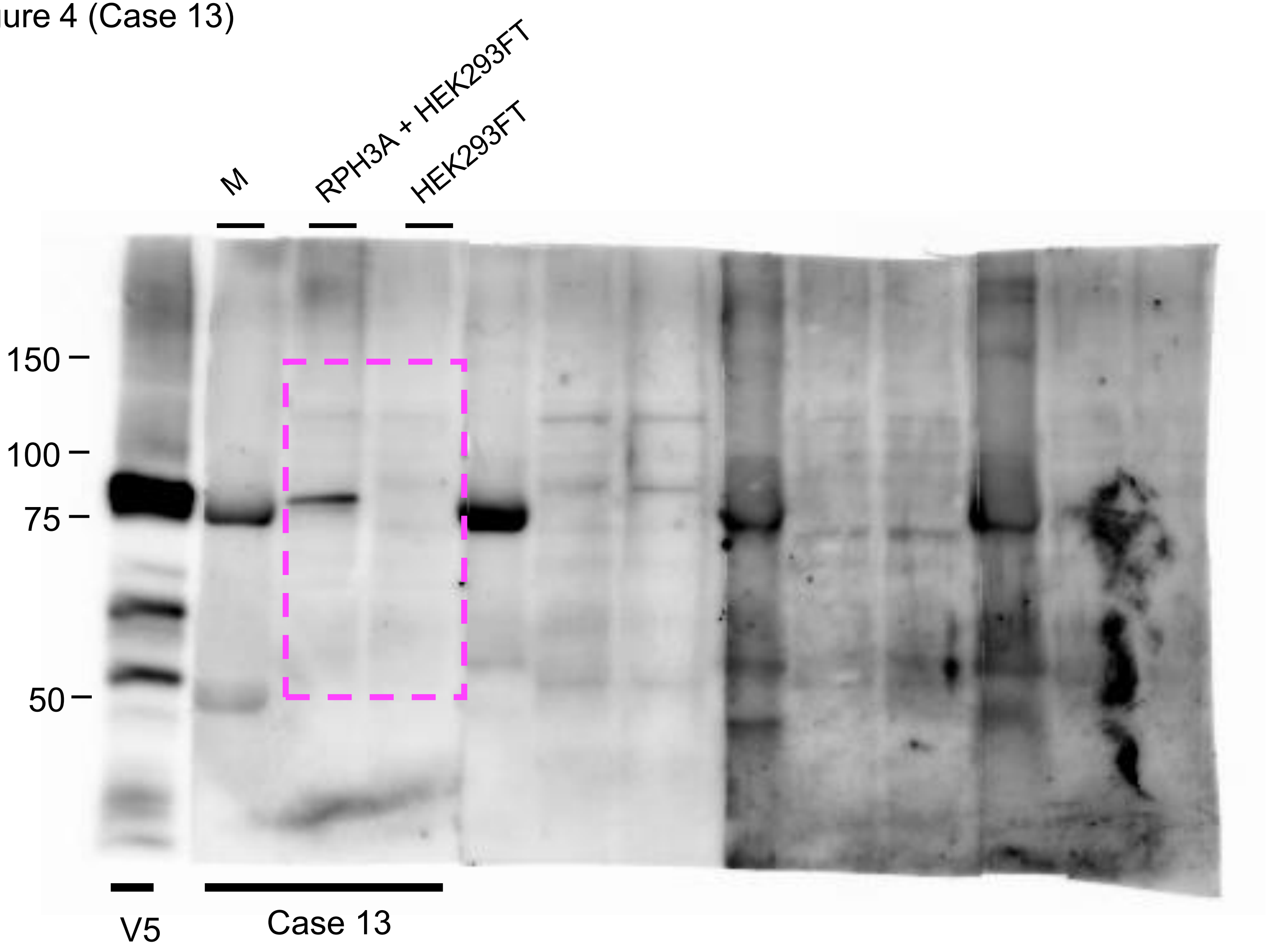

Supplementary figure 15

Original image of figure 4 (Case 14)

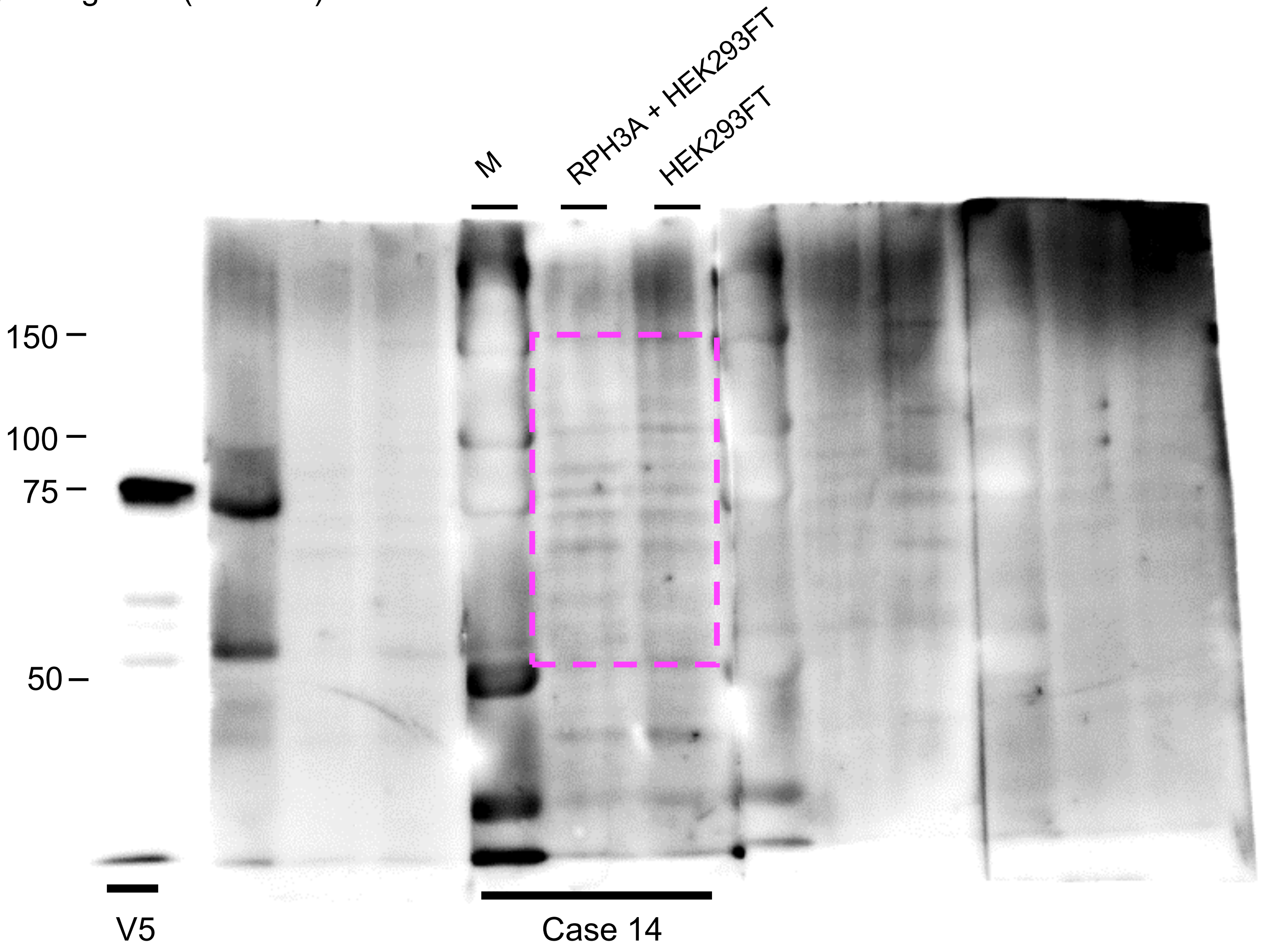

Supplementary figure 16

Original image of figure 4 (Case 15)

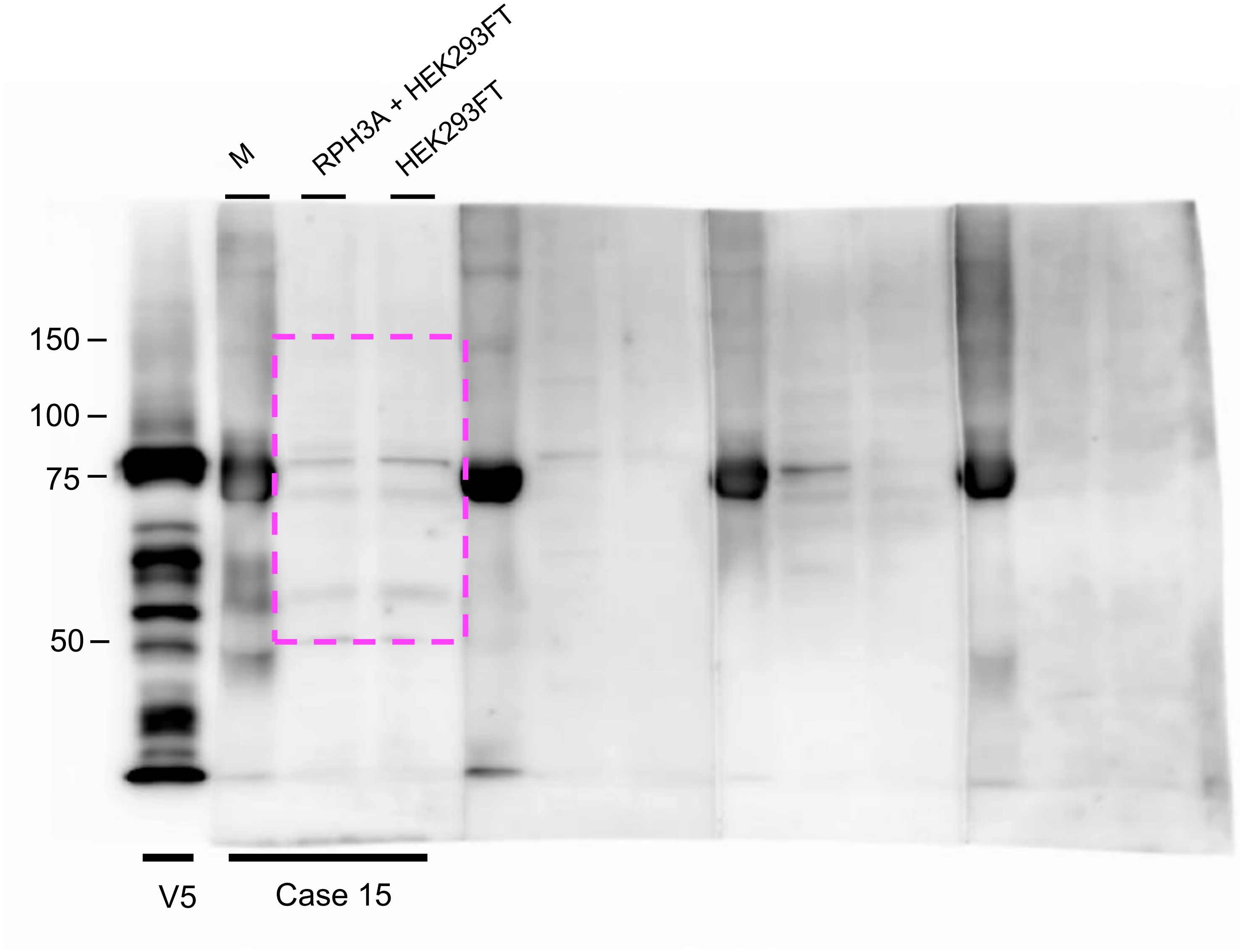

Supplementary figure 17

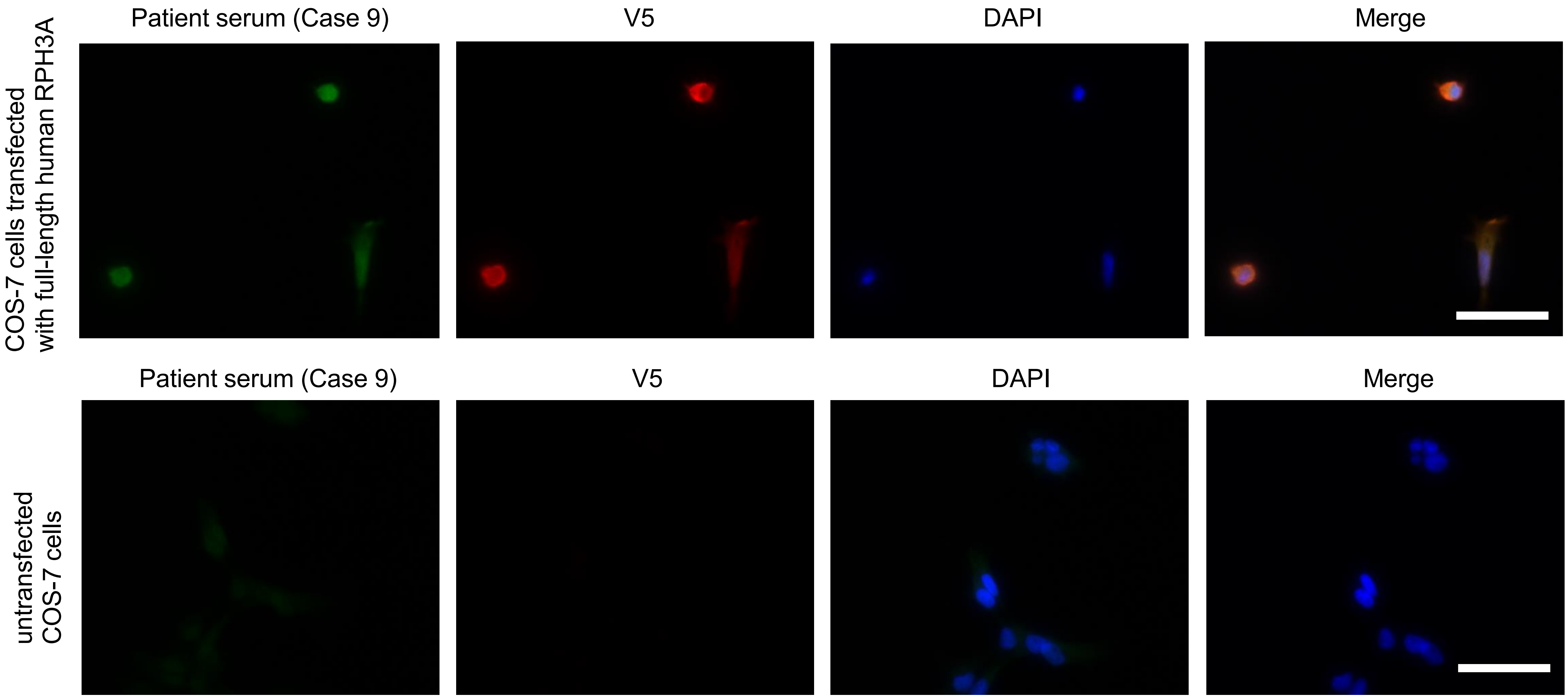

Supplementary figure 18

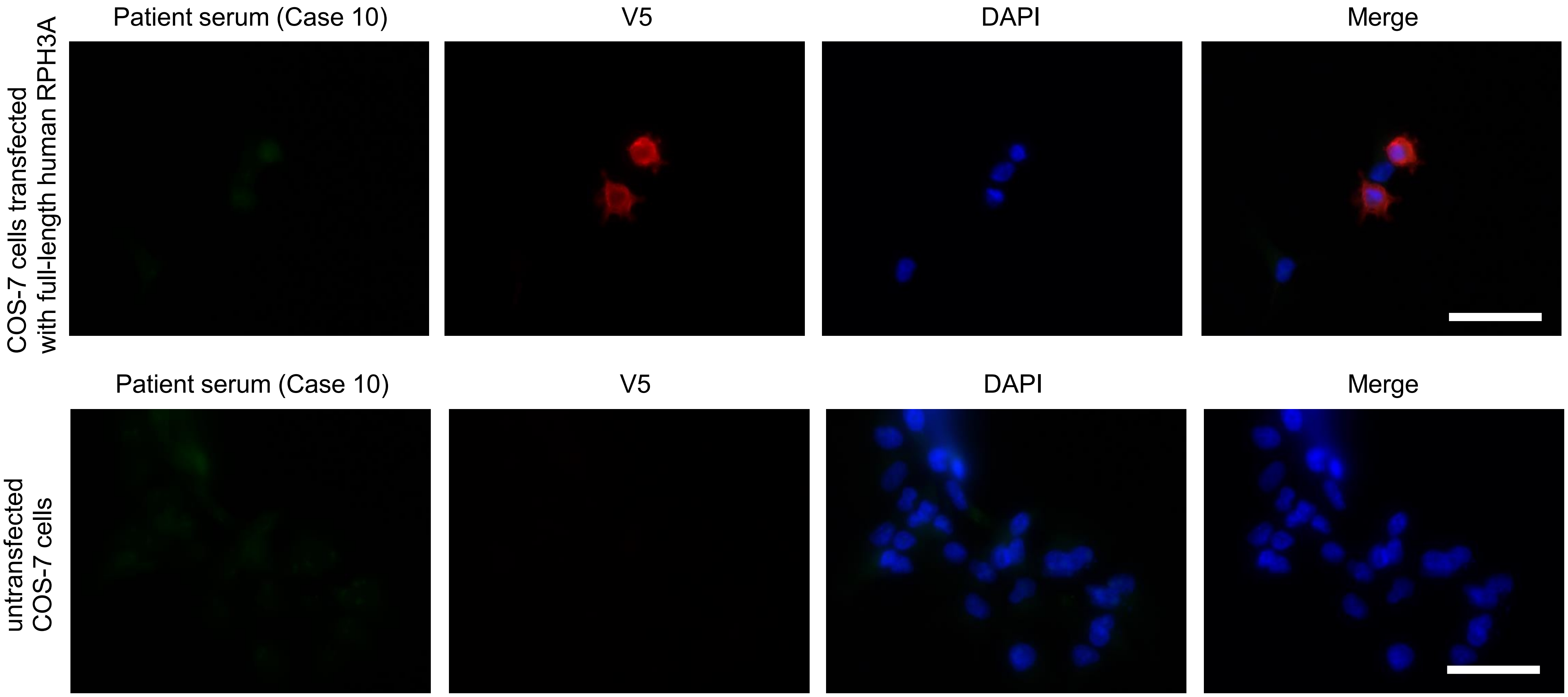

Supplementary figure 19

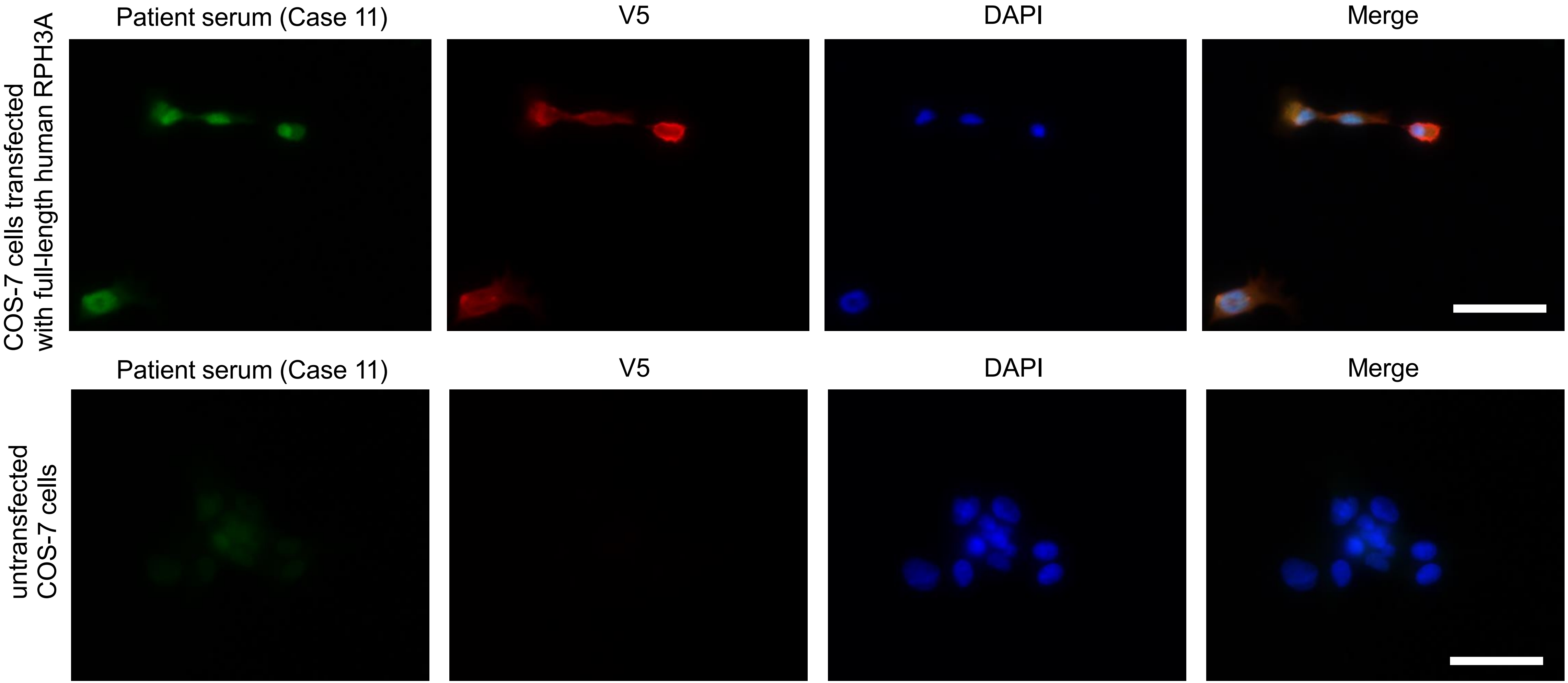

Supplementary figure 20

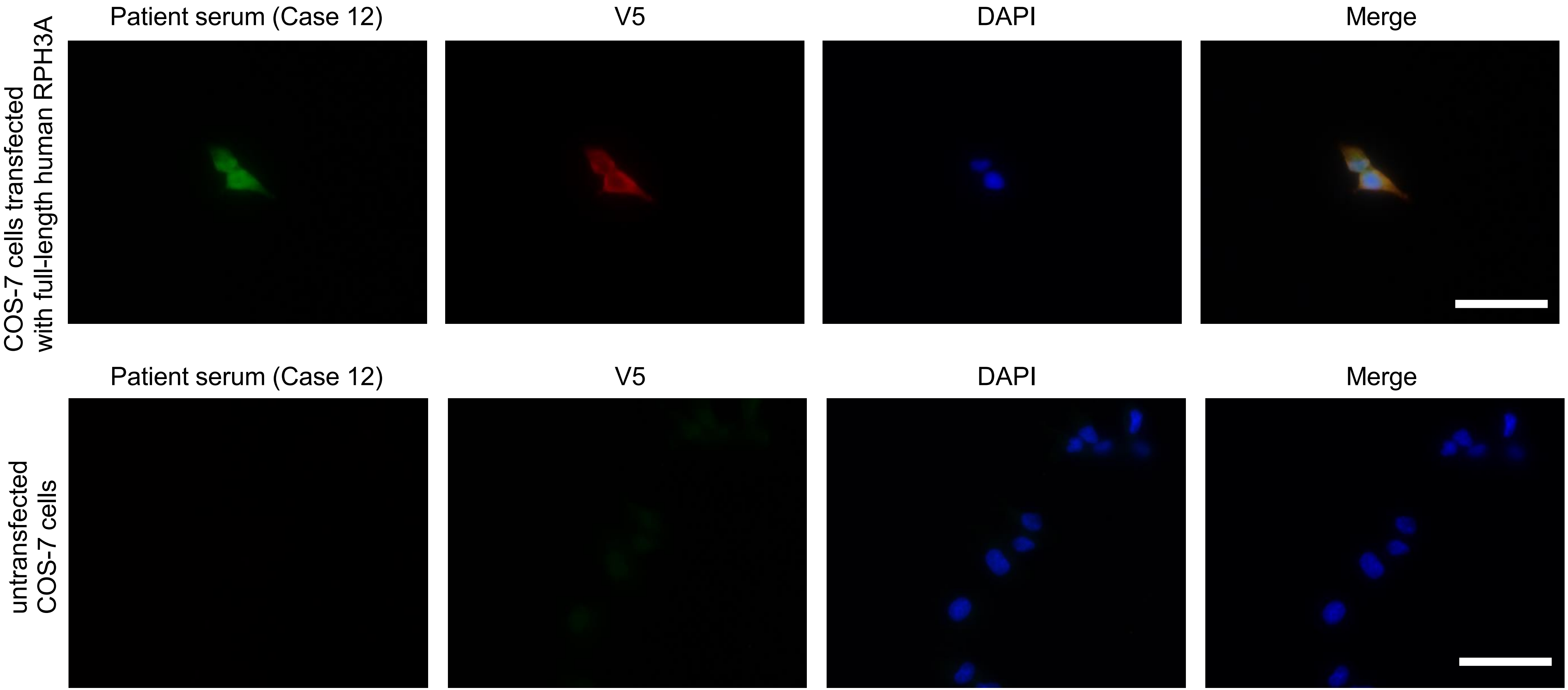

Supplementary figure 21

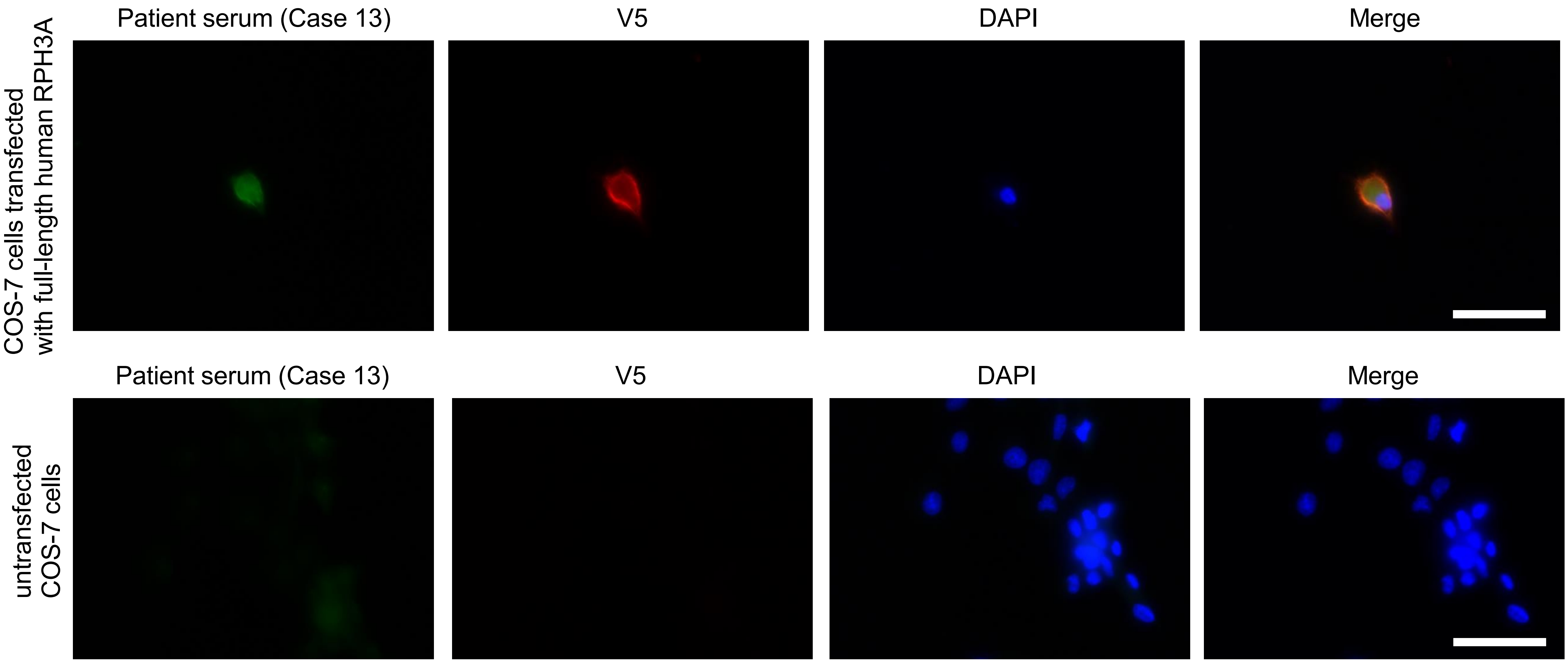

Supplementary figure 22

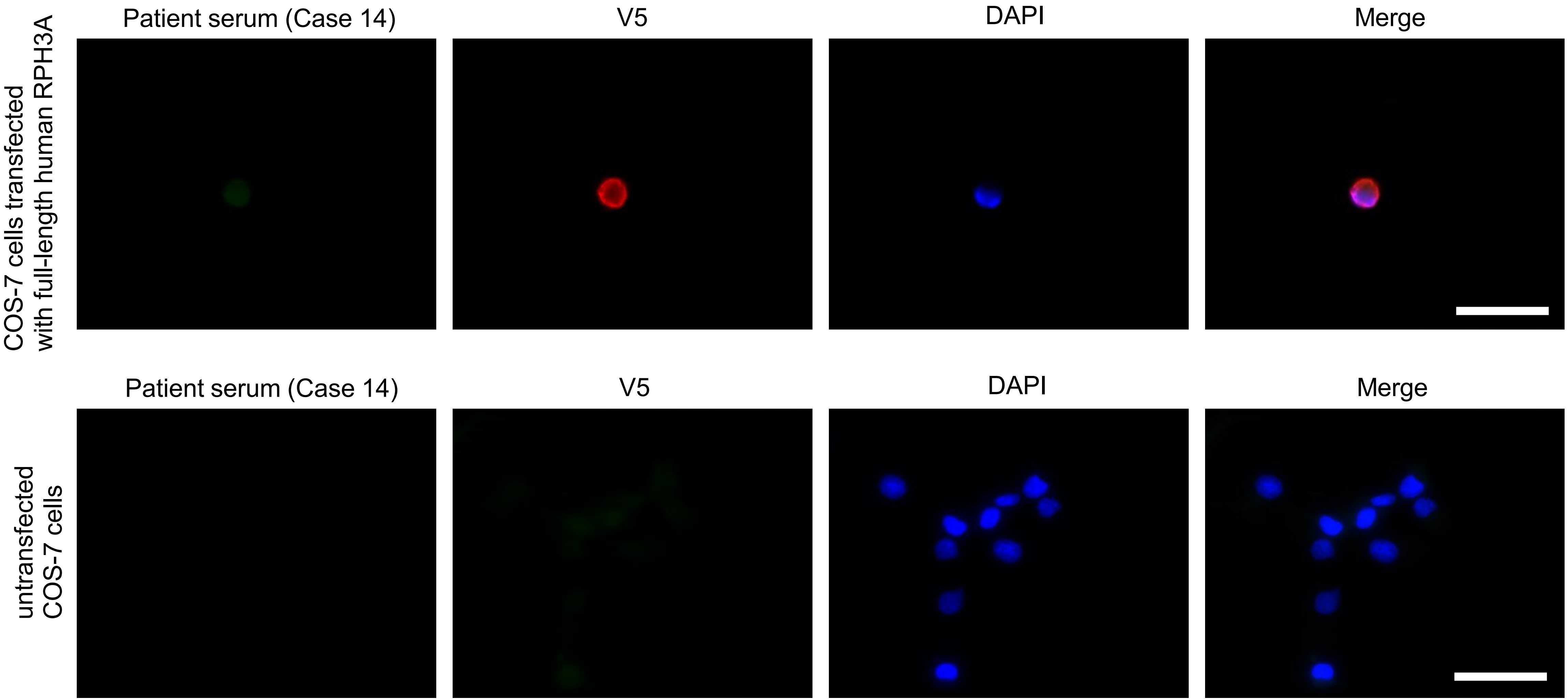

Supplementary figure 23

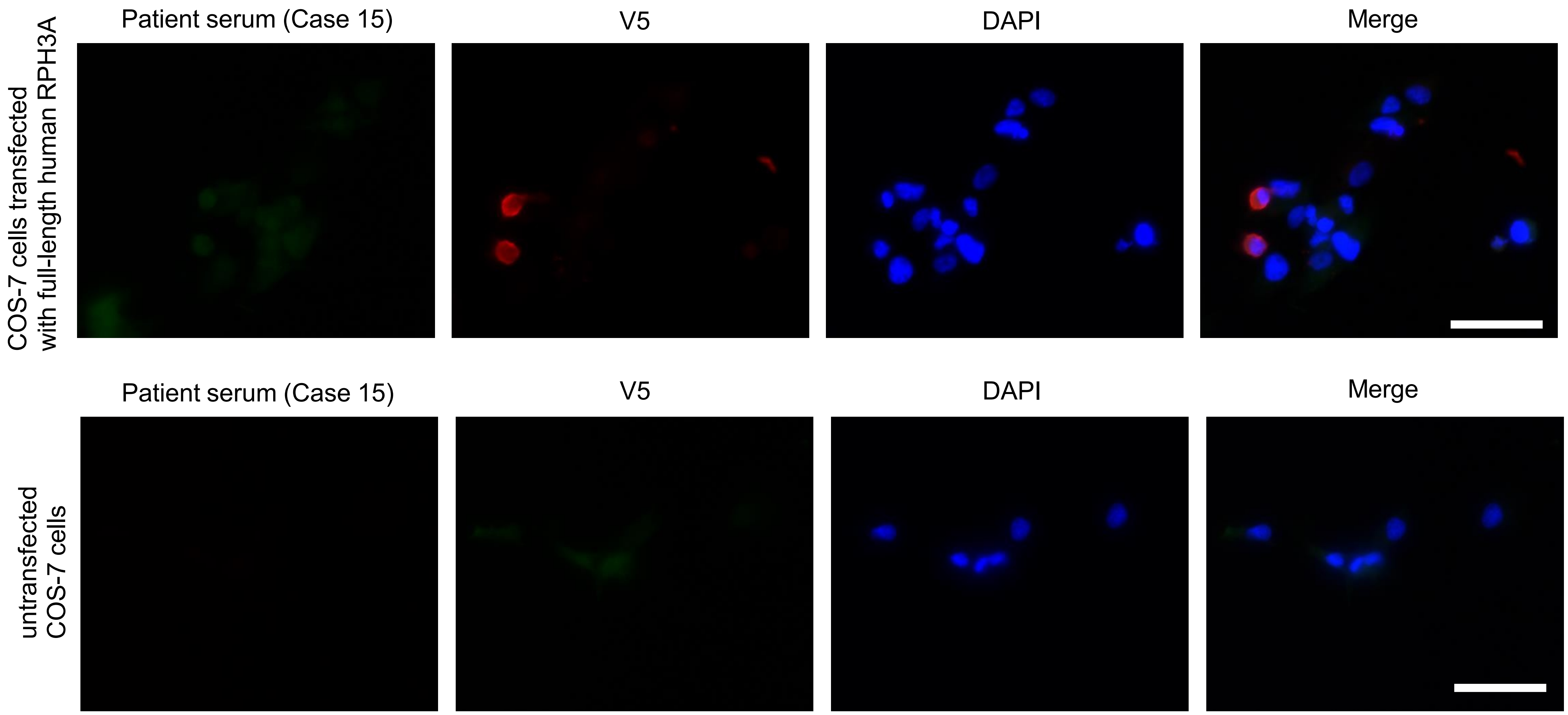

Supplementary figure 24

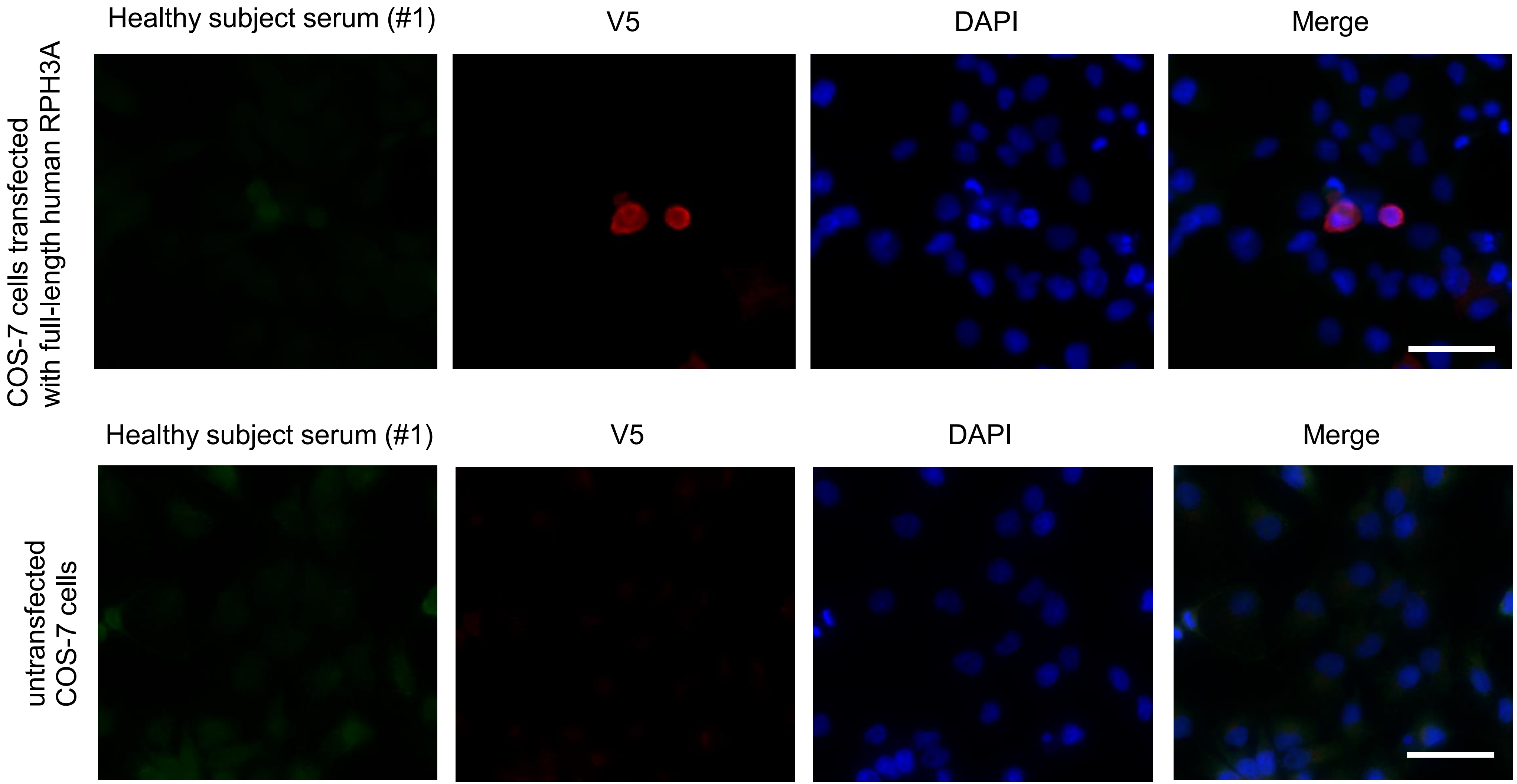

Supplementary figure 25

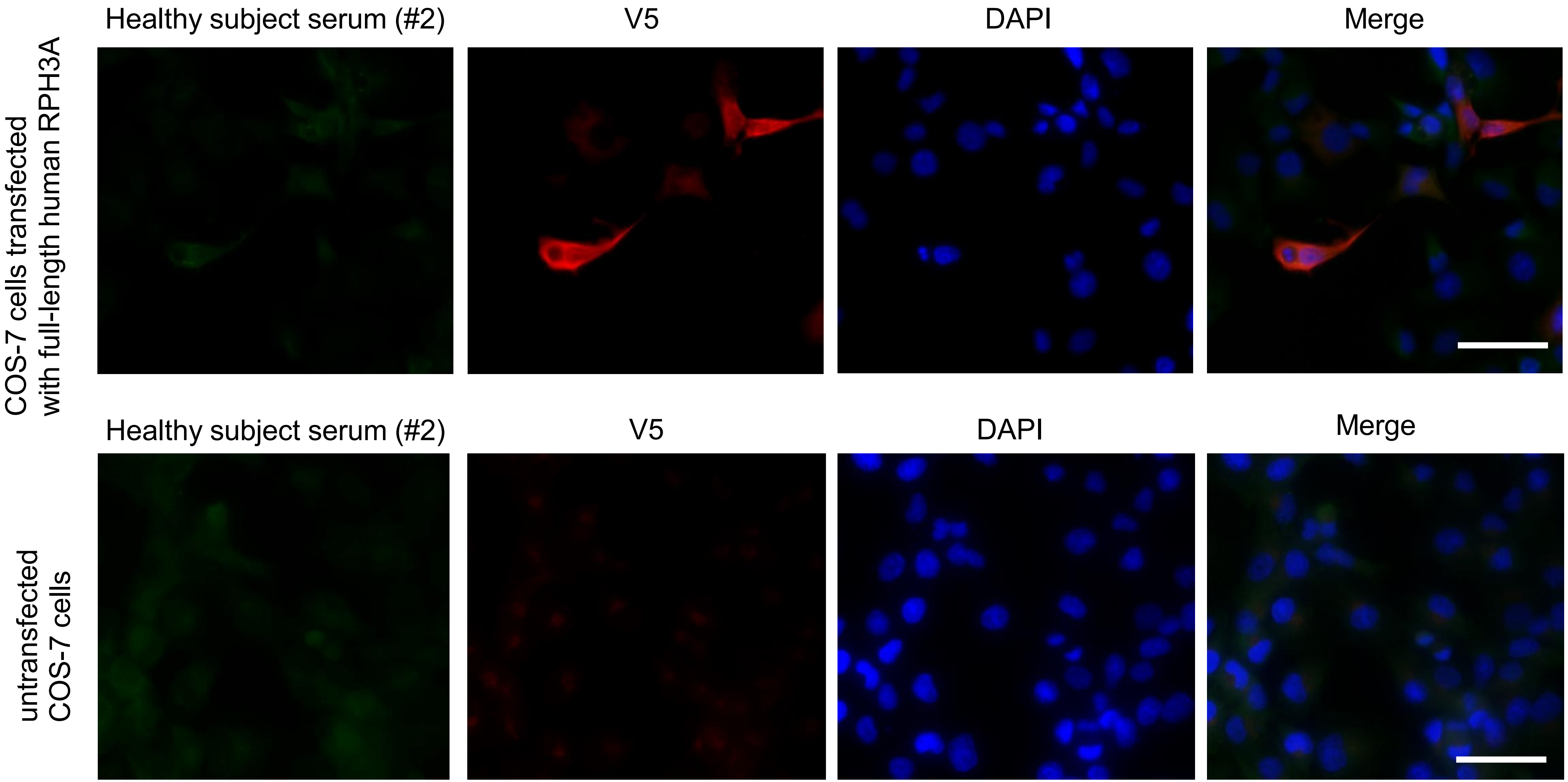

Supplementary figure 26

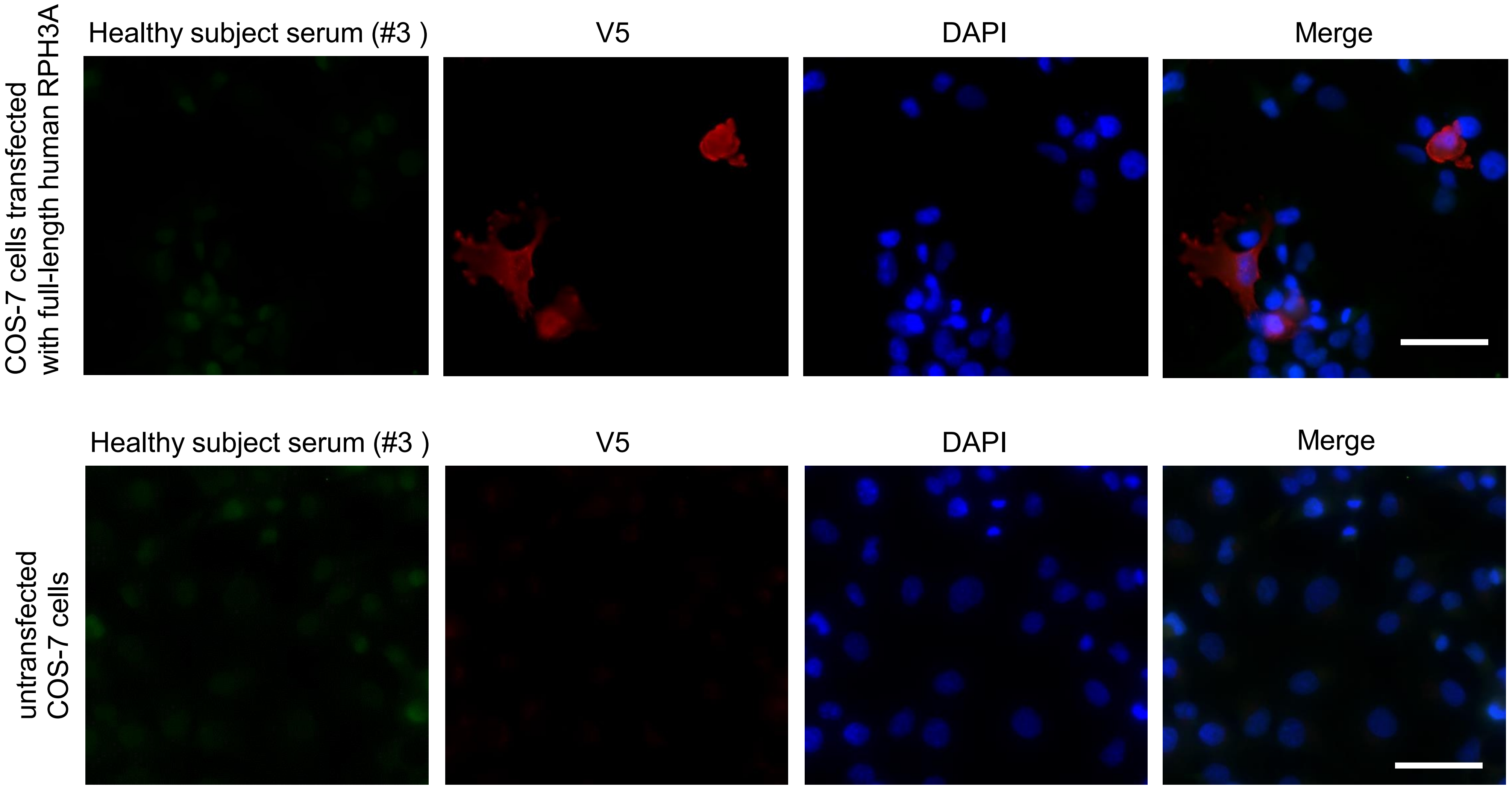

Supplementary figure 27

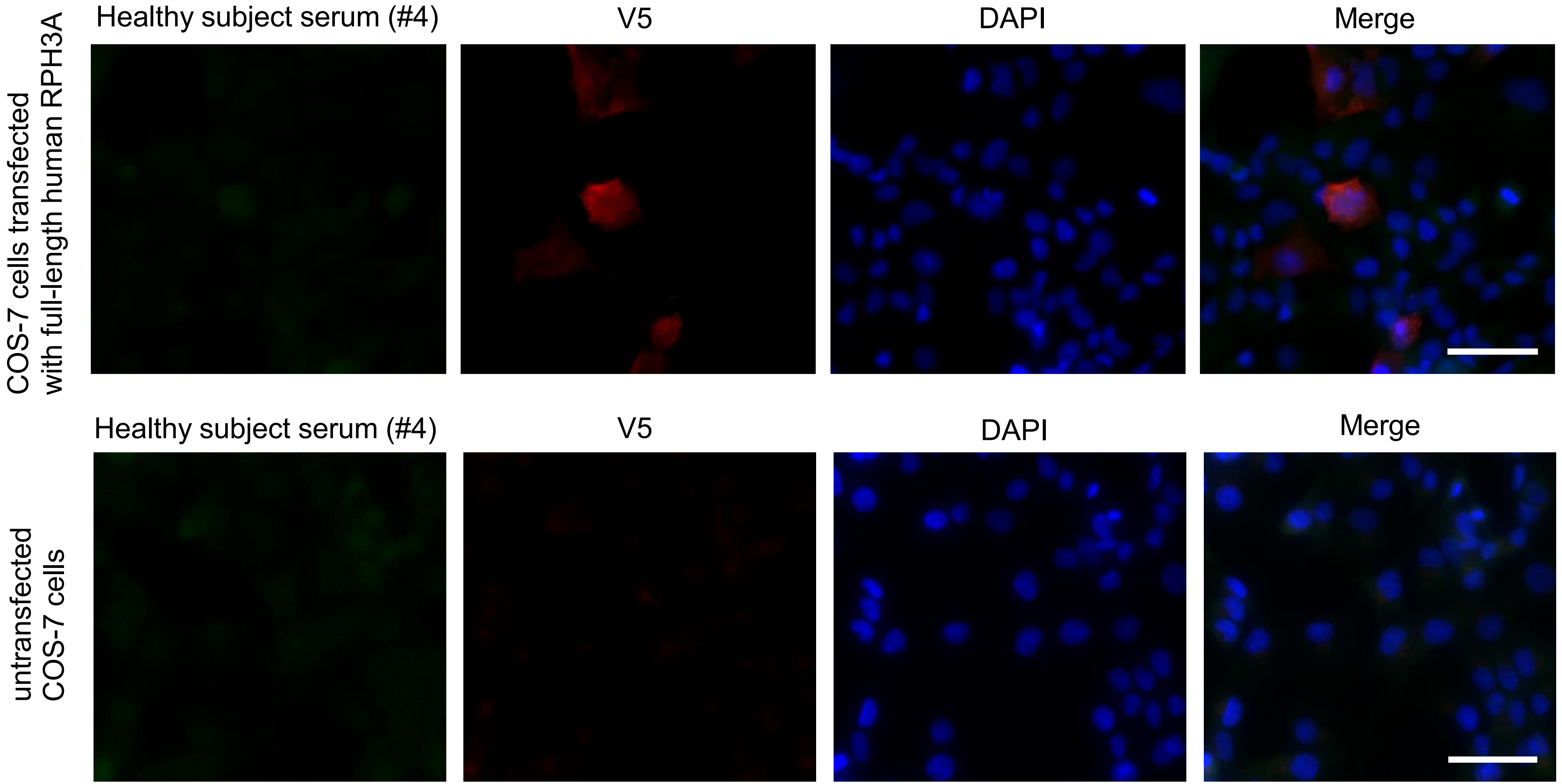

Supplementary figure 28

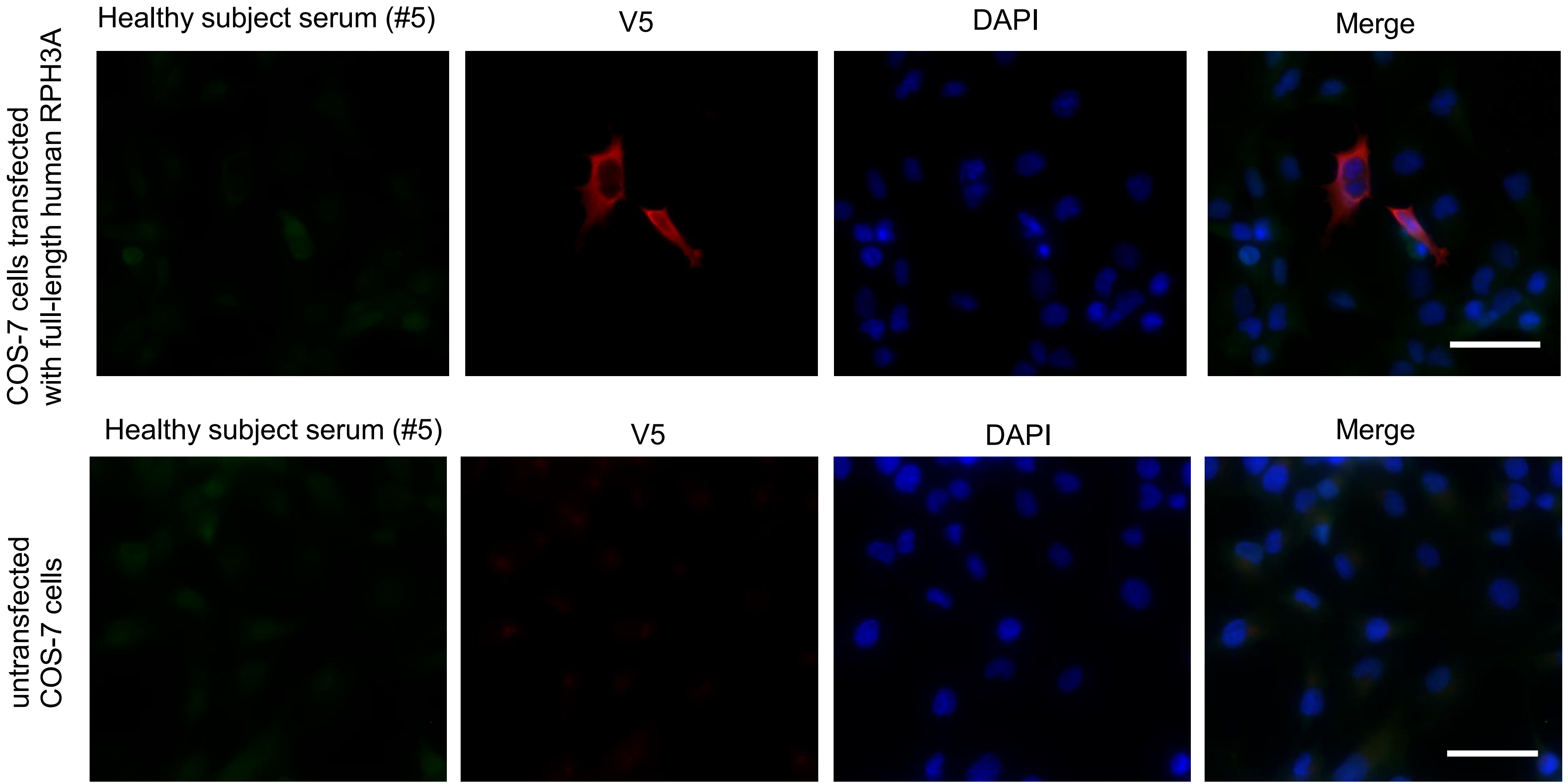

Supplement: Supplementary file 1 — Supplementary Information. [file 41598_2022_8552_MOESM1_ESM.pdf]
